# A novel ketogenic diet that reduces seizures and prevents liver steatosis leads to related gut microbiome changes and restores cecal short-chain fatty acid levels in the rapid kindling rat model of epileptogenesis

**DOI:** 10.1080/29933935.2025.2567677

**Published:** 2025-10-09

**Authors:** Hester Meeusen, Julia Lohr, Tiemen van Eijndthoven, Rozemarijn S. Kalf, Guus Roeselers, Ardy van Helvoort, Jan A. Gorter, Erwin A. van Vliet, Sebastian Tims, Jose P. Silva, Eleonora Aronica

**Affiliations:** a Department of (Neuro)pathology, Amsterdam UMC Location University of Amsterdam, Amsterdam Neuroscience, Amsterdam, The Netherlands; b Danone Research & Innovation, Utrecht, The Netherlands; c Institute of Nutrition and Translational Research in Metabolism, Maastricht University Medical Center, Maastricht University, Maastricht, The Netherlands; d Center for Neuroscience, Swammerdam Institute for Life Sciences, University of Amsterdam, Amsterdam, The Netherlands; e Danone Global Research & Innovation Center B.V., Utrecht, The Netherlands

**Keywords:** Ketogenic diet, epilepsy, fatty liver, gut microbiome, short chain fatty acids, saturated fatty acids, stearic acid, omega-3 polyunsaturated fatty acids, fibers

## Abstract

We recently introduced an innovative ketogenic diet (KD) demonstrating superior antiseizure efficacy in a rat kindling model of epileptogenesis without inducing hepatic steatosis compared to a classic KD. This new diet features reduced fat content, lower plasma ketosis levels, and incorporates new ingredients, including a novel fermentable fiber blend, omega-3 polyunsaturated fatty acids (PUFAs) that partially replace omega-6 PUFAs, and medium-chain fatty acids that partially substitute for stearic acid. In the present study, we conducted gut microbiota analyses on cecum samples to elucidate its contribution to these effects. The analysis revealed distinctive features compared to the classic KD and control diet: higher weighted alpha diversity, distinct beta diversity, increased *Bifidobacterium* and *Clostridium sensu stricto 1* abundances, and altered genera abundances correlated with seizure scores and liver triglyceride content. Furthermore, these genera abundances were linked to stearic acid and omega-3 PUFA plasma levels. Notably, the novel KD restored short-chain fatty acid levels and the butyrate-to-propionate ratio in the cecum, possibly driving lipid oxidation and ketone production while preventing liver steatosis. These findings suggest the involvement of the gut microbiome in mediating the antiseizure efficacy and reducing the adverse metabolic effects of a KD, thereby enhancing its utility for epilepsy treatment.

## Introduction

Increasing preclinical evidence suggests that the composition of the gut microbiome and its capacity to interact with the host may influence health status. The high-fat (~45 kcal%) “Western style” diet is associated with a (possibly irreversible) reduction in alpha diversity, elevated chronic inflammation and metabolic disease.^1^ Likewise, the high-fat (~90 kcal%) low-carbohydrate ketogenic diet (KD) reportedly lowers alpha diversity, and the associated changes in microbiota abundances have been proposed to protect against obesity and metabolic disorders.^2^ The KD is an effective treatment option for various neurological conditions, particularly for drug-resistant epilepsy.^3^ Ancillary changes in the composition and function of the microbiome have been proposed as one of the mechanisms that drives the protective effect of KD in seizure suppression.^4-6^

One proposed mechanism through which the gut microbiome may influence local and systemic physiology is through the release of specific metabolites into the gut lumen and their local and systemic distribution.^7^ For example, short-chain fatty acids (SCFAs) released by the gut microbiota locally constitute the main energy source of the gut epithelium and may confer a range of beneficial metabolic health benefits, including protection against obesity, diabetes mellitus, cardiovascular disease, and liver disease.^8^ SCFA and other microbial gut metabolites may also have neuroprotective properties, such as lowering neuroinflammation.^8^ In the hippocampal kindling mouse model of temporal lobe epilepsy, butyrate was found to block epileptogenesis.^9^ However, SCFA production in a KD is limited,^10^ with one study showing 55% reduction in overall SCFA and 20% reduction in butyrate production in epilepsy patients after one month of treatment with the classic 4:1 KD.^11^ Butyrate and increasing the butyrate-to-propionate ratio boosts ketone production in hepatocytes.^12^ A loss in butyrate or a reduction in the butyrate to propionate ratio could limit ketone production^12^ and the health benefits of the classic KD; therefore, ingredients for the KD are sought that favorably alter the production of SCFAs by the gut microbiota.

To mitigate the drastic loss of SCFA production, we added a dietary fiber blend favoring butyrate over propionate production in a human *in vitro* fermentation assay to a novel KD variant with a reduced fat content (1.9:1 fat to protein plus carbohydrate ratio). Moreover, saturated long-chain fatty acids, particularly stearic acid, were exchanged for medium-chain fatty acids and omega-3 PUFAs.

The novel KD showed increased effectiveness in delaying behavioral seizure progression compared to the classic rodent-adapted 6:1 KD and control diet in the hippocampal rapid kindling rat model of epileptogenesis. Furthermore, the novel KD improved metabolic health, with normalized liver triglyceride levels and normalized free fatty acid plasma levels compared to the 6:1 classic KD.^13^

In the present study, we aimed to clarify whether the novel KD rescued SCFA production by the gut microbiota. A second aim of the study was to explore the possible link between changes in the gut microbiota composition and therapeutic efficacy of the novel KD by correlating the abundance of bacterial genera with (1) plasma ketone levels, (2) seizure scores, (3) liver triglyceride content, and (4) free fatty acid plasma concentrations.

To meet these aims, we measured SCFA levels in the cecum and profiled the gut microbiome by 16s rRNA gene sequencing in the cecum of kindled and nonkindled rats exposed to either a control diet, a novel KD or a classic rodent-adapted 6:1 KD in our previous study.^13^ These assessments were correlated with the phenotypic outcomes reported in previous and present studies.^13^

## Materials and methods

### Diets and study design

The full diet composition and experimental design are detailed in a previous publication,^13^ from which cecal samples were used for this study. In short, the experimental diets included the control diet (according to BioServ AIN-93M formulation containing 50.9 g/kg cellulose), novel KD (formulation by Danone Research & Innovation, containing 48.8 g/kg cellulose, 23.6 g/kg inulin, 23.6 g/kg galacto oligosaccharides, 12.6 g/kg soy fiber, and 2.6 g/kg resistant starch) and classic KD (according to BioServ F3666 formulation, containing 47.7 g/kg cellulose) and were provided daily in plastic feeding bowls.^13^ The animals were exposed to the diets for three weeks, starting 5 d before the onset of kindling. Initially, we had an *n* = 18 per group, and after dropout, the group numbers were as follows: control diet *n* = 15, novel KD *n* = 16, and classic KD *n* = 14. Meanwhile in the nonkindled groups, there were no dropouts (control diet *n* = 18, novel KD *n* = 18). All animals that did not drop out were included in the present study. The animals were kindled for 4 d, and behavioral assessments were performed during the third week of the experiment. At sacrifice, the animals were ad libitum fed, and specimens were collected, including the cecum, which was stored at −80 °C. Only *ex vivo* measurements were performed for the present study; animal husbandry, procedures, and ethical licensing are reported in ref. ^13^.

### Cecal pH measurement

pH levels were determined by applying a glass microelectrode pH meter (SevenMultiTM equipped with glass electrode, InLab® Micro, Mettler Toledo, Tiel, the Netherlands) directly into the cecum sample.

### SCFA, lactate, and ammonia analyses

Cecum samples were diluted 1:10 in ice-cold phosphate-buffered saline (PBS) and homogenized by adding glass beads (3 mm in diameter) followed by vigorous vortexing for 5 min at maximum speed (Multi Reax, Heidolph Instruments, Schwabach, Germany). The supernatant was centrifuged for 3 min, at 15,000 × *g* at 4 °C. Two hundred  microliters of clear supernatant was used for SCFA analysis. The SCFAs propionic, acetic, n-butyric, isobutyric, n-valeric, and isovaleric acids were quantified using a Shimadzu-GC2025 gas chromatograph and flame ionization detector including hydrogen as the mobile phase. Sample concentrations were calculated using a calibration curve generated by an internal standard 2-ethylbutyric acid. For lactate determination, the samples were first centrifuged for 10 min at 16000 × *g* at 4 °C, followed by enzyme inactivation at 100 °C for 10 min and cleared using 10 min, 16000 × *g* centrifugation. D- and L-lactate dehydrogenase were determined enzymatically using an L-lactic acid detection kit (Boehringer Mannheim, Mannheim, Germany). To measure ammonia, an ammonia (rapid) assay kit (Cat. Number K-AMIAR Megazyme, Neogen, Ayr, Scotland) was used. Before ammonia was measured with this kit, the samples were fixed with trichloroacetic acid (TCA, 30% w/v) to a final concentration of 3% TCA per sample.

### Cecal DNA extraction

The DNA extraction from ~200 µg of cecum sample was performed using QIAamp Fast DNA Stool Mini Kit (Qiagen, Hilden, Germany). 300 mg, 0.1 mm glass beads and 1 ml InhibiteEX Buffer were added to each sample, followed by a bead-beating step (FastPrep-24 instrument, MP Biomedicals, Eschwege, Germany) for 30 s, program 5.5. Additional steps were conducted according to the manufacturer's instructions. In brief, after heat treatment at 95 °C for 5 min, the supernatant was collected by centrifugation, and lysis was performed in Buffer AL and proteinase K at 70 °C for 10 min. The total volume was transferred to a QIAamp spin column after ethanol (96%–100%) was added to the lysate. After centrifugation, the DNA was attached to the QIAamp membrane, followed by two wash steps with buffers AW1 and AW2. Elution was performed by adding 100 µl of nuclease-free water (not DEPC-treated) (Invitrogen/Thermo Fisher Scientific, Bleiswijk, the Netherlands) and incubated for 1 min at room temperature followed by 1 min of centrifugation at 20000 × *g* at room temperature.

### 16S rRNA gene sequencing & bioinformatics analyses

The V3‒V4 region of the 16S rRNA gene was PCR-amplified with the primers Bact-0341F (5′-CCTACGGGNGGCWGCAG-3′) and Bact-0785R (5′-GACTACHVGGGTATCTAATCC-3’)^14^ and sequenced on the MiSeq platform (Illumina, Eindhoven, the Netherlands) as described previously^15^ using the 2 × 300 bp paired-end protocol. Sequencing was performed by Danone Research and Innovation. In each sequencing run, 5% of PhiX (Illumina, Eindhoven, the Netherlands) was included as an internal control. The read pairs were demultiplexed and trimmed (*q* > 20) before being merged using Paired-End reAd meRger (PEAR)^16^ v0.9.2. Merged reads with *q* > 25 over a window of 15 bases, no ambiguous bases, and a minimal length of 300 were retained. These were dereplicated and counted using mothur v.1.41.1,^17^ and reads with a low abundance (fewer than 2 reads across all samples) were discarded. Chimeras were removed using VSEARCH v.2.3.4^18^ with the ChimeraSlayer reference database.^19^ Reads that contained PhiX or adapters as defined in Deblur v.1.1.0^20^ (part of QIIME2^21^) were eliminated. Taxonomic assignment was performed using the RDP classifier v.2.2^22^ against the SILVA v. 138^23^ database. Reads with eukaryotic or chloroplast assignments, as well as reads with a low relative abundance of up to 0.0005% in all the samples, were excluded from further downstream analysis. Rarefaction was applied to the taxa by phyloseq^24^ and vegan packages^25^ in R v4.0.2 (a language and environment for statistical computing https://www.R-project.org/ (2018)) in order to perform alpha diversity calculations using the Chao1 and Shannon index metrics. The beta diversity was computed over all samples using the vegan R v4.0.2 package and bray distance.

### Plasma fatty acid analysis

Blood samples were collected after three weeks on the experimental diets, and the subsequent plasma fatty acid analysis was further detailed in a previous study.^13^ The total omega-3 to omega-6 PUFA plasma concentration ratio was calculated by dividing the total omega-3 fatty acid concentrations (C18:3, C18:4, C20:3, C20:5, C22:3, C22:5, and C22:6) by the total omega-6 fatty acid concentrations (C18:2, C18:3, C20:2, C20:3, C20:4, C22:4, and C22:5).

### Statistical analysis

After testing for statistical differences and adjusting the *p*-value, as specified in the sections below, a *p*-value < 0.05 was considered significant for all the statistical tests applied to the sequencing data. The statistical significance of the differences in alpha diversity was assessed with the pairwise_wilcox_test function from the rstatix package in R v4.0.2^26^ followed by Benjamini‒Hochberg *p*-value adjustment. The statistical significance of differences in beta diversity was assessed using the permutation ANOVA function adonis2 from the package vegan in R. Where applicable, a post hoc pairwise adonis was applied.^27^ To investigate the homogeneity of the samples within each diet group, betadisper from the R package vegan^25^ was used, followed by a Tukey honest significant differences test.

Genera with a minimum mean relative abundance of 0.5% across all samples were tested for differential abundance between study arms. Differential abundance was performed with generalized linear models with mixed effects on sequencing counts, using the glmmTMB package v 1.1.2.3 in R v4.0.2,^28^ followed by Anova.glmmTMB, applying the chi-square test for significant differences. The resulting *p*-values were corrected using Benjamini–Hochberg. Models containing significant interaction effects were followed up with a post hoc using the emmeans package v 1.7.5^29^ in R. For visualization purposes, low-abundance taxa were transformed with the natural log.

The composition plot at the phylum level contains the arithmetic mean of all samples belonging to one group. At the genus level, on average, the top 20 most abundant taxa for each sample are individually shown. The correlation between kindled samples and genera with a minimum mean relative abundance of 0.5% across all samples was computed on the natural log-transformed relative abundances and visualized using the ComplexHeatmap package (v. 2.10.0)^30^ with Euclidean distance and average clustering.

Statistical differences in physiological measurements (such as pH value, ammonia concentration, short-chain fatty acid concentration, and fatty acid concentration) between groups were assessed using the pairwise Wilcox test function from the rstatix package followed by Benjamini‒Hochberg *p*-value adjustment. Measurements of the physiological correlations between the relative abundance of genera with a minimum mean relative abundance of 0.5% across all samples, and the physiological measurements were performed using the cor.test function with Spearman's rho.

## Results

### Alpha and beta diversity

**Figure 1.**
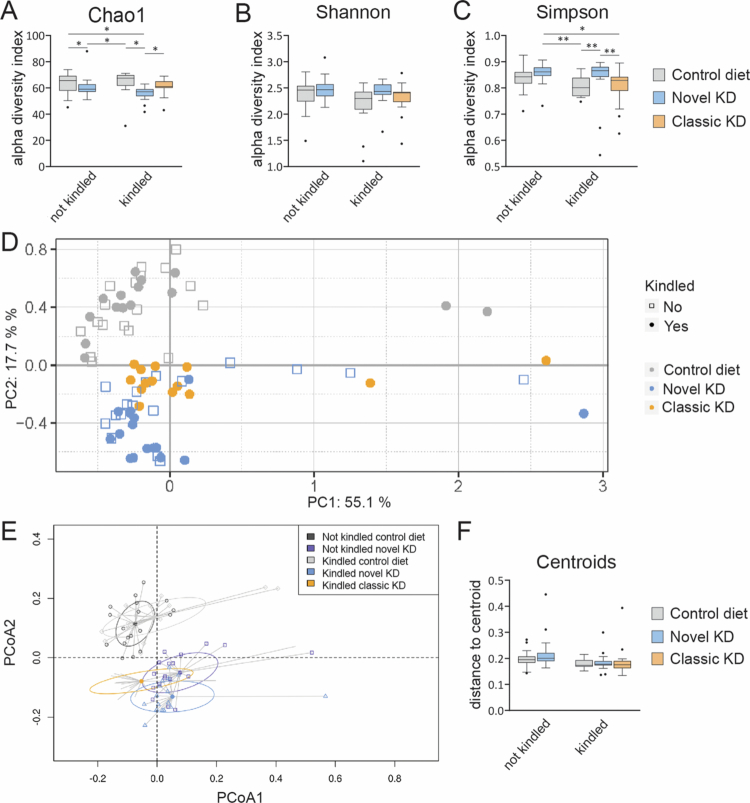
Alpha and beta diversity. Box and whisker plots showing the alpha diversity using the unweighted Chao1 (A), weighted Shannon (B), and weighted Simpson indices (C). PCA by Bray–Curtis showing the degree of dissimilarity between samples for all five groups showed distinct clusters for the diet groups, without a separation based on kindling (D). Beta diversity was homogeneous between the kindled diet groups, as shown in a PCoA plot with one centroid for each group (E) and box and whisker plots of distances to each group's centroid (F). The dots in (A), (B), (C), and (F) represent outliers; *p < 0.05, **p < 0.01.

Alpha and beta diversity were analyzed to determine the median microbiota diversity and evenness for each group, and the degree of dissimilarity between the different groups, respectively. Alpha diversity was analyzed using three indices, the Chao1 index, which considers all genera equally irrespective of their relative abundances, and the Shannon and Simpson indices, which lend greater weight to the more abundant genera and, respectively, less or no weight to rare genera in the sample. First, the effect of kindling was investigated for the control diet and novel KD groups. No significant effects were found (*p* = 0.61, unkindled control diet vs kindled control diet, Chao 1 index; *p* = 0.12, unkindled novel KD vs kindled novel KD, Chao 1 index; Figure 1A; *p* = 0.42, unkindled control diet vs kindled control diet, Shannon index; *p* = 0.85, unkindled novel KD vs kindled novel KD, Shannon index; Figure 1B; *p* = 0.07, unkindled control diet vs kindled control diet, Simpson index; *p* = 0.67, unkindled novel KD vs kindled novel KD, Simpson index; Figure 1C). Therefore, subsequent alpha diversity assessments focused on comparing only the effect of the diets on the kindled groups.

The unweighted Chao1 index showed a reduction in alpha diversity for the novel KD group compared to the control diet and classic KD groups (*p* = 0.016, kindled control diet vs kindled novel KD; *p* = 0.021, kindled novel KD vs kindled classic KD; Figure 1A), indicating a reduced number of genera in this group. Meanwhile, the Shannon index did not reveal differences between the groups (*p* = 0.21, kindled control diet vs kindled novel KD; *p* = 0.59, kindled novel KD vs kindled classic KD; Figure 1B). The weighted Simpson index showed an increase in alpha diversity in the novel KD group compared to the control diet and classic KD groups (*p* = 0.003, kindled control diet vs kindled novel KD; *p* = 0.007, kindled novel KD vs kindled classic KD; Figure 1C), indicating an increase in microbiota evenness with the novel KD compared to the control diet and classic KD groups when very low-abundance genera are less or not considered. Alpha diversity was not significantly affected by the classic KD (*p* = 0.088, kindled control diet vs kindled classic KD, Chao 1 index; *p* = 0.59, kindled control diet vs kindled classic KD, Shannon index; and *p* = 0.617, kindled control diet vs kindled classic KD, Simpson index).

To visualize the closeness in the gut microbiota composition at genera level between groups, also called beta diversity, a principal component analysis (PCA) based on Bray‒Curtis dissimilarity was conducted. Since cecum samples were collected from three cohorts in each group, the first step in the analysis was to determine whether cohort formation was a confounding factor. Cohorts did not significantly differ by Bray–Curtis PCA analysis (*F*_2_ = 1.59, *p* = 0.073). In a follow-up analysis that distinguished between diet and kindling effects, a clear separation between the diet groups was observed (permutation ANOVA, *F*_2_ = 14.0, *p* < 0.001; Figure 1D), while there was no kindling effect (*F*_1_ = 1.49, *p* = 0.15). In contrast to the absence of a kindling effect on alpha diversity according to any of the above-mentioned statistical measures, Bray‒Curtis PCA analysis detected an interaction between diet and kindling on beta diversity (*F*_1_ = 2.36, *p* = 0.023), which post hoc analysis revealed was found between the kindled groups of one diet versus the unkindled groups of a different diet. Since cross-kindling–cross-diet interactions were outside the focus of the present study, they were not considered for further analysis.

To determine the homogeneity of each group, centroids were plotted for each group using Principal Coordinate Analysis (PCoA, Figure 1E). There were no differences in distances to each group's centroid, meaning that the groups showed a uniform degree of similarity (*F*_4,76_ = 0.562, *p* = 0.71; Figure 1F).

### Cecum microbial community distribution

**Figure 2.**
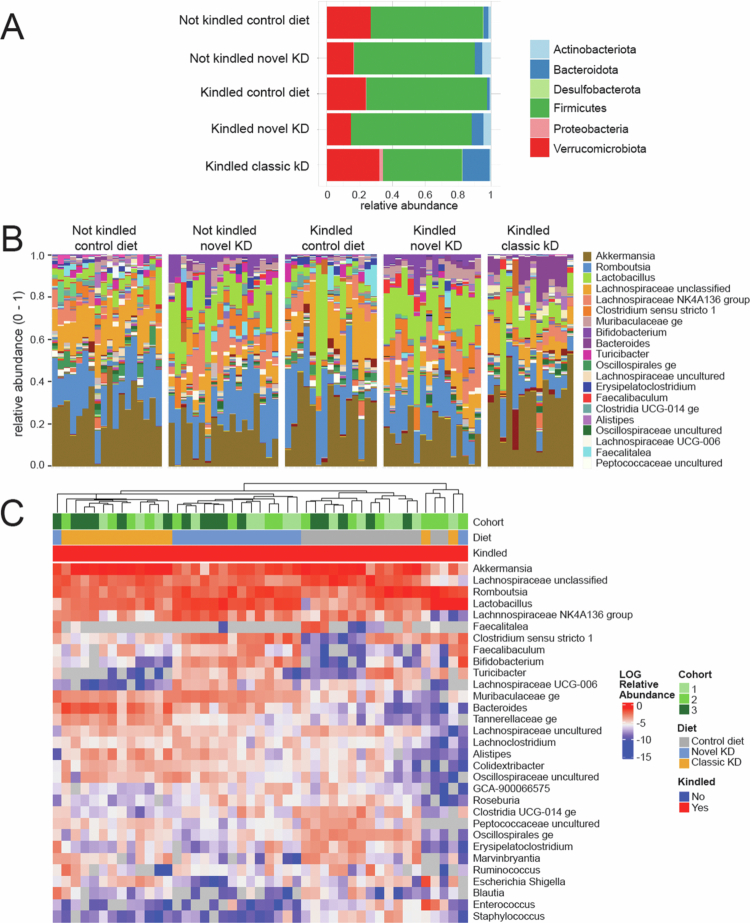
Cecum microbial community structure. Composition plots showing the taxonomic distributions of bacteria from cecum 16S rRNA gene sequencing at the phylum level for the five groups (A), genus level for the five groups (B), and split by individual sample. (C) Hierarchical clustering of individual samples, including cohort, diet, and all genera, with a minimum abundance of 0.5%.

The phylum-level analyses showed that the overall distribution of the microbiota at the phylum level was affected mainly by diet (Figure 2A). Among the kindled groups, the novel KD increased the relative contribution of Actinobacteriota (*p* < 0.001) and Bacteroidota (*p* < 0.001) compared to the control diet. The classic KD increased the relative abundance of Bacteroidota (*p* < 0.001) and Proteobacteria (*p* = 0.005), and Desulfobacterota (*p* = 0.005) at the expense of Firmicutes (*p* < 0.001) compared to the control diet. When comparing the two KDs, the novel KD increased the relative abundances of Actinobacteriota (*p* < 0.001) and Firmicutes (*p* < 0.001), whereas the abundances of Bacteroidota (*p* = 0.009), Proteobacteria (*p* < 0.001), Verrucomicrobiota (*p* = 0.003), and Desulfobacterota (*p* = 0.014) were lower.

Within each group, the microbiota composition at the genus level showed some variability between samples (Figure 2B). Hierarchical clustering of similarities in genera abundances, separated the samples by diet groups (Figure 2C) and not by cohorts of kindled versus non-kindled groups (Supplementary Figure S1), further confirming the absence of kindling effects on the gut microbiota composition. All further analyses focused therefore on the kindled groups.

### Differentially abundant genera

**Figure 3.**
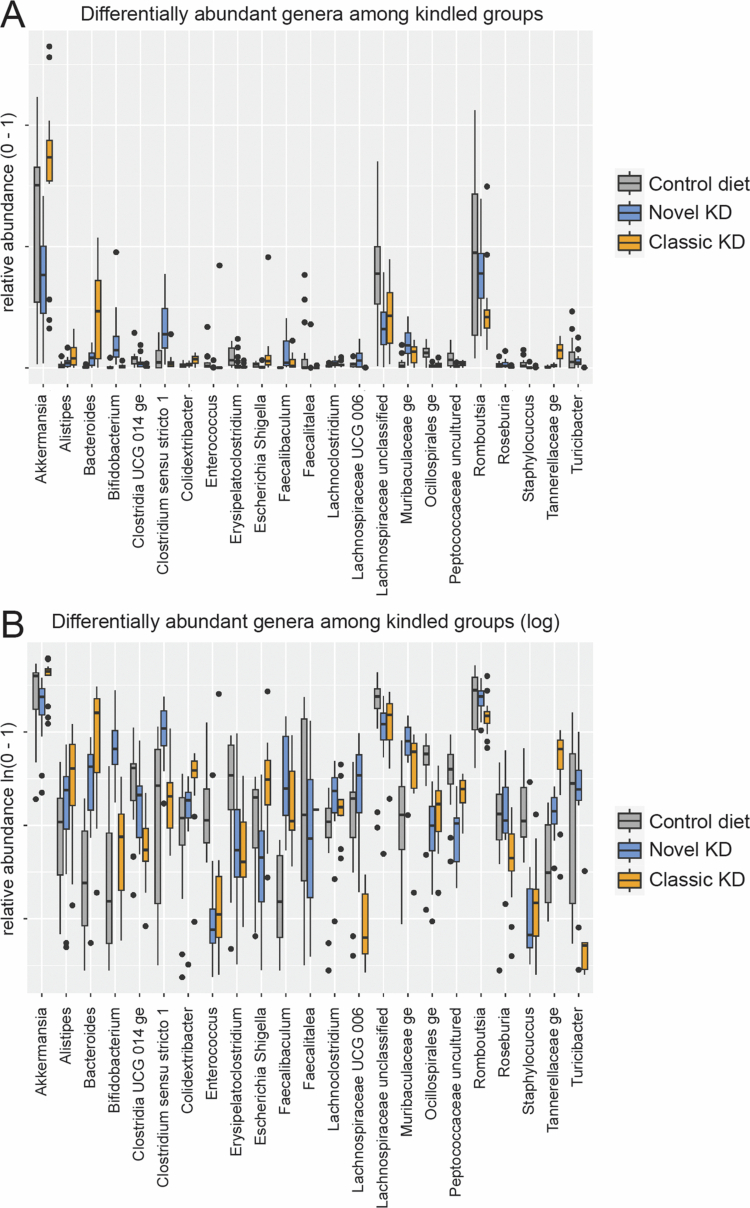
Differentially abundant genera. (A) Box and whisker plots for differentially abundant genera in the kindled groups on a scale from 0 to 1, with the total abundance set to 1. (B) Box and whisker plots on a natural log scale to visualize differences between groups for genera with a relatively low abundance. Dots represent outliers. Statistical differences of the chi-squared test are depicted in Table 1.

Among the kindled groups, 23 genera were differentially abundant (Figure 3A and B). The adjusted *p*-values of each pairwise comparison and the fold-changes are shown in Table 1. Genera whose abundance was uniquely increased by the novel KD comprised *Bifidobacterium* (*p* < 0.0001, vs classic KD and control diet) and *Clostridium sensu stricto I* (*p* = 0.022 vs control diet; *p* < 0.0001 vs classic KD), in contrast *Peptococcaceae uncultured* was uniquely decreased (*p* < 0.0001 vs control diet; *p* = 0.034 vs classic KD).

**Table 1.** Differentially abundant genera: pairwise comparisons, adjusted *p*-values, and fold-changes.

	Novel KD vs control diet		Classic KD vs novel KD		Classic KD vs control diet	
	Fold-change	Adjusted *p*-value	Fold-change	Adjusted *p*-value	Fold-change	Adjusted *p*-value
*Akkermansia*		0.147	2.3	0.004		0.381
*Alistipes*		0.163		0.071	6.7	0.002
*Bacteroides*	42.0	<0.0001	5.7	<0.0001	238.6	<0.0001
*Bifidobacterium*	298.3	<0.0001	0.1	<0.0001		0.713
*Clostridia UCG 014 ge*		0.159	0.2	<0.0001	0.1	<0.0001
*Clostridium sensu stricto 1*	6.2	0.022	0.1	<0.0001		0.357
*Colidextribacter*		0.584	2.6	0.028	4.9	0.033
*Enterococcus*	0.02	<0.0001		0.19		0.208
*Erysipelatoclostridium*		0.159		0.109	0.1	<0.0001
*Escherichia Shigella*		0.056	12.5	<0.0001	1.8	0.004
*Faecalibaculum*	81.7	<0.0001		0.109	27.1	<0.0001
*Faecalitalea*		0.35		0.471	0.0	0.011
*Lachnoclostridium*	2.7	0.018		0.302		0.103
*Lachnospiraceae UCG 006*		0.072	0.003	<0.0001	0.01	<0.0001
*Lachnospiraceae unclassified*	0.4	0.022		0.43		0.113
*Muribaculaceae ge*	11.8	<0.0001		0.08	8.4	0.024
*Oscillospirales ge*	0.1	<0.0001		0.318	0.2	0.001
*Peptococcaceae uncultured*	0.2	<0.0001	2.5	0.034		0.202
*Romboutsia*		0.652	0.5	0.019	0.4	0.041
*Roseburia*		0.682	0.3	0.001	0.2	0.012
*Staphylococcus*	0.03	<0.0001		0.995	0.1	<0.0001
*Tannerellaceae ge*	7.2	0.036	7.4	<0.0001	52.8	<0.0001
*Turicibacter*		0.424	0.0	<0.0001	0.0	<0.0001

Genera whose abundance was increased by both KDs, compared to the control diet included *Bacteroides* (*p* < 0.0001), *Faecalibaculum* (*p* < 0.0001), *Muribaculaceae ge* (*p* < 0.0001, novel KD; *p* = 0.024, classic KD), and *Tannerellaceae ge* (*p* = 0.036, novel KD; *p* < 0.0001, classic KD) with a higher increase of *Bacteroides* and *Tannerellaceae ge* in the classic KD than in the novel KD (*p* < 0.0001). Lowered abundances were found for both KDs, compared to the control diet in the genera *Oscillospirales ge* (*p* < 0.0001, novel KD; *p* < 0.001, classic KD) and *Staphylococcus* (*p* < 0.0001).

Genera, whose abundances were uniquely increased by the classic KD included *Colidextribacter* (*p* = 0.028 vs novel KD; *p* = 0.033 vs control diet) and *Escherichia shigella* (*p* < 0.0001 vs novel KD; *p* = 0.004 vs control diet), while uniquely lower abundances were observed for *Clostridia UCG 014 ge* (*p* < 0.0001, vs novel KD and control diet), *Lachnospiraceae UCG 006* (*p* < 0.0001 vs novel KD and control diet), *Romboutsia* (*p* = 0.019 vs novel KD; *p* = 0.041 vs control diet), *Roseburia* (*p* = 0.001 vs novel KD; *p* = 0.012 vs control diet), and *Turicibacter* (*p* < 0.0001 vs novel KD and control diet).

### Cecum SCFA, metabolites, and pH levels

**Figure 4.**
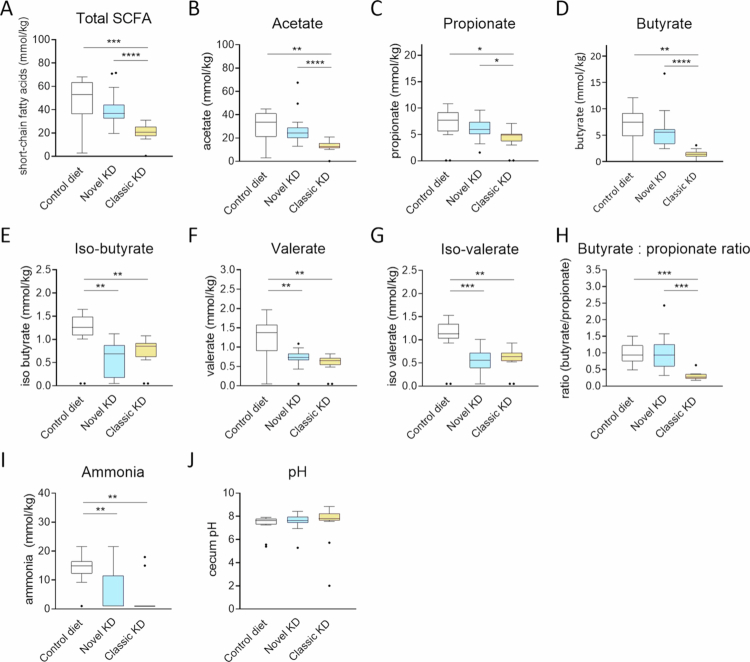
Cecum microbiota metabolites and cecum pH among kindled animals. Box and whisker plots of the cecum median total SCFA (A), acetate (B), propionate (C), butyrate (D), isobutyrate (E), valerate (F), and isovalerate levels (G), the resulting butyrate-to-propionate molar ratio (H), box and whisker plots of ammonia levels (I), and pH values (J). Metabolite values are expressed in mmol/kg, and values below the detection range are set to the lowest limit of detection and were excluded from the butyrate-to-propionate ratio analysis. Dots represent outliers. Significance was assessed using pairwise Wilcoxon test, *p < 0.05, **p < 0.01, ***p < 0.001, and ****p < 0.0001.

The classic KD drastically reduced overall SCFA cecum levels (*p* = 0.0009 vs control diet). Treatment with the novel KD restored SCFA levels to those comparable to the control diet group (*p* = 0.28) and higher than those in the classic KD group (*p* < 0.0001 vs classic KD; Figure 4A). The classic KD reduced SCFA levels compared to the control diet, including acetate (*p* = 0.001; Figure 4B), propionate (*p* = 0.011; Figure 4C), and butyrate (*p* = 0.002; Figure 4D). Furthermore, the branched short-chain fatty acids isobutyrate (*p* = 0.001; Figure 4E) and isovalerate (*p* = 0.001; Figure 4G) as well as valerate (*p* = 0.002; Figure 4F), which are unbranched saturated fatty acids with five-carbon chains, were lower in the classic KD group compared to the control diet group. The acetate and butyrate levels were reduced more (62% and 81%, respectively, compared to control diet) than the propionate, isobutyrate, and isovalerate levels (36%, 33% and 43%, respectively), resulting in a decreased butyrate to propionate ratio in the classic KD (*p* = 0.0006 vs the control diet; Figure 4H). The novel KD rescued acetate (*p* = 0.31 vs control diet; *p* < 0.0001 vs classic KD; Figure 4B), propionate (*p* = 0.21 vs control diet; *p* = 0.03 vs classic KD; Figure 4C), and butyrate cecum levels (*p* = 0.21 vs control diet; *p* < 0.0001 vs classic KD; Figure 4D), but not a reduction in isobutyrate (*p* = 0.001 vs control diet; *p* = 0.34 vs classic KD; Figure 4E), isovalerate (*p* = 0.001 vs control diet; *p* = 0.36 vs novel KD; Figure 4G), and valerate cecum levels (*p* = 0.003 vs control diet; *p* = 0.06 vs novel KD; Figure 4F). By elevating butyrate cecum levels more than propionate cecum levels, the novel KD normalized the butyrate-to-propionate cecum molar ratio (*p* = 0.89 vs control diet; *p* = 0.0006 vs classic KD; Figure 4H).

Cecum ammonia levels were reduced by both novel KD (*p* = 0.004) and classic KD (*p* = 0.004) compared to the control diet and were the same between the novel KD and classic KD (*p* = 0.38; Figure 4I). pH measurements did not differ between the groups (*p* = 0.66, control diet vs novel KD; *p* = 0.46, novel KD vs classic KD; and *p* = 0.28, control diet vs classic KD; Figure 4J). There were no differences in ammonia, lactate or pH levels between the kindled and nonkindled groups on the same experimental diet (Supplementary Figure S2).

### Correlations between genera abundances and phenotypic outcomes

**Figure 5.**
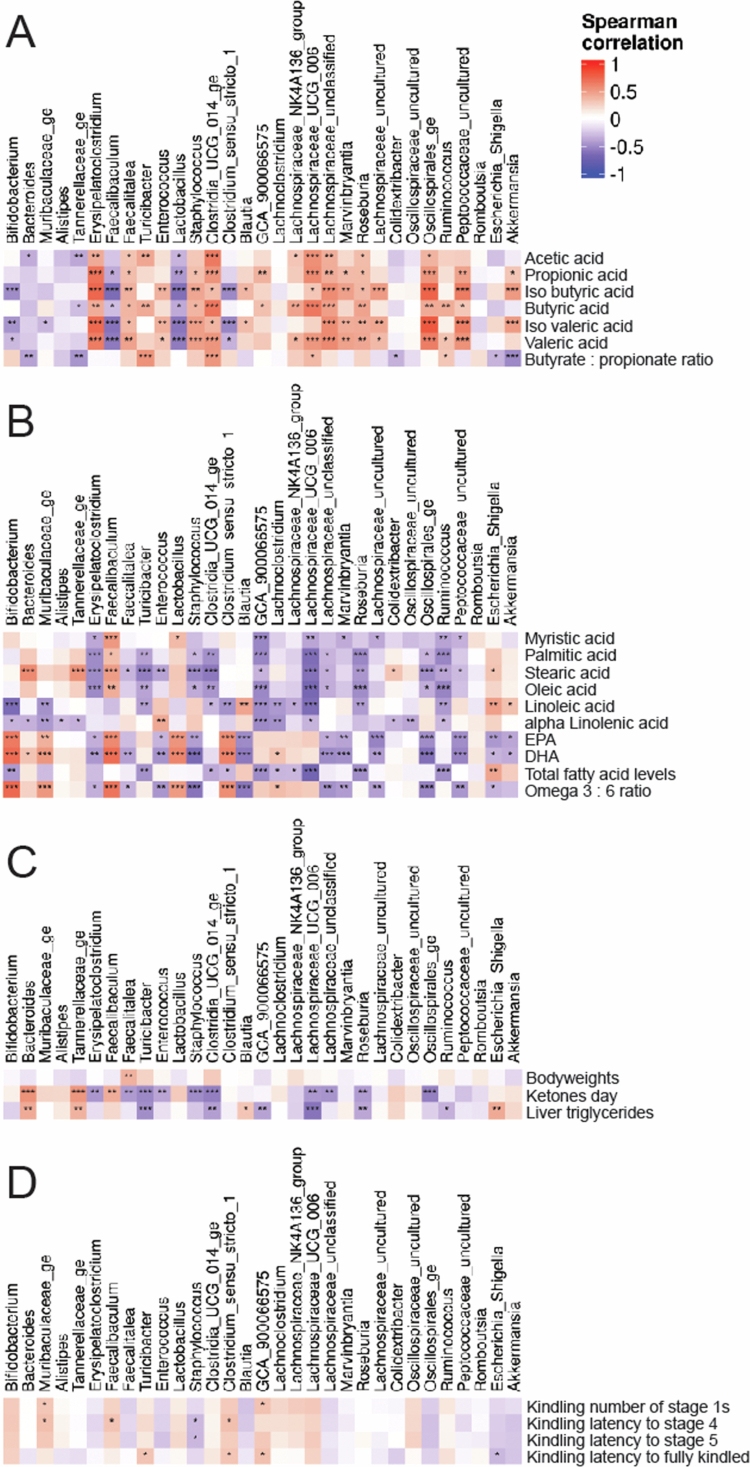
Spearman correlation analysis of microbiota abundance with phenotypic outcomes. Spearman correlation plots of genera abundance with metadata, including (A) cecum SCFA levels and the butyrate-to-propionate ratio; (B) single plasma fatty acid levels (myristic acid, palmitic acid, stearic acid, oleic acid, linoleic acid, *α*-linolenic acid, EPA, and DHA), the total plasma fatty acid levels, and the total omega-3 fatty acid to omega-6 fatty acid ratio; (C) body weights, blood ketone levels, and liver triglyceride levels; (D) rapid kindling seizure load parameters, including the number of Racine stages 1 experienced, latency to reach Racine seizure stages 4 and 5, and latency to reach the fully kindled stage. Spearman correlation adjusted *p*-values are indicated in the correlation plots, *p < 0.05, **p < 0.01, and ***p < 0.001.

To identify genera that may influence SCFA production, plasma ketone levels, plasma lipid levels, liver triglyceride content, and seizure severity in the rapid rat kindling study,^13^ Spearman correlation coefficients and their significance values for correlating genera abundances with these phenotypic measures were calculated (Figure 5A–D; Supplementary Tables S1, S2). To relate only to genera abundances and increase the power, samples from all three diet groups were pooled for the analysis.

### Correlations with SCFA cecum levels

Genera positively correlated with acetate, butyrate, and propionate cecum levels included *Clostridia UCG 014 ge*, *Roseburia*, *Oscillospirales ge*, *Lachnospiraceae unclassified*, *Erysipelatoclostridium*, *Faecalitalea, Turicibacter*, *GCA 900066575*, and *Peptococcaceae uncultured* (Figure 5A; Supplementary Tables S1, S2). Five of these genera, *Clostridia UCG 014 ge, Roseburia, Faecalitalea, Erysipelatoclostridium*, and *Turicibacter,* were downregulated by the classic KD but not the novel KD compared to the control diet (Table 1). Meanwhile, the novel KD downregulated only three of these genera, *Oscillospirales ge*, *Lachnospiraceae unclassified*, and *Peptococcaceae uncultured*. Furthermore, the abundance of two genera, *Fecalibaculum* and *Lactobacillus*, was negatively correlated with the acetate, butyrate, and propionate cecum levels, of which *Fecalibaculum* abundance was increased in both KDs. When comparing the two KDs, the genera that were increased in abundance for the novel KD compared to the classic KD and positively correlated with cecum acetate and butyrate were *Turicibacter, Clostridia UCG 014 ge, Lachnospiraceae UCG 006*, and *Roseburia*, indicating that these genera may have been involved in the restoration of butyrate production in the novel KD. Finally, the butyrate-to-propionate cecum molar ratio correlated positively with the *Clostridia UCG 014 ge*, *Ruminococcus,* and *Turicibacter* abundances, whereas no negative correlations were noted. Among these genera, the abundances of *Clostridia UCG 014 ge* and *Turicibacter* were downregulated in the classic KD group. In summary, the classic KD downregulated more genera positively correlated with SCFA cecum levels than the novel KD, while they both upregulated only one genus, *Faecalibaculum*, which was negatively correlated with SCFA cecum levels.

### Correlations with plasma ketone concentrations

Plasma ketone concentrations were positively correlated with *Bacteroides*, *Tannerellaceae* ge, and *Faecalibaculum*. All of these genes were upregulated primarily by the classic KD, except for *Fecalibaculum*, which was higher with the novel KD. Furthermore, they correlated negatively with *Turicibacter*, *Roseburia*, *Lachnospiraceae UCG 006*, *Lachnospiraceae unclassified*, *Enterococcus*, and *Faecalitalea*. Four of these genera, *Turicibacter*, *Roseburia*, *Lachnospiraceae UCG 006*, and *Faecalitalea*, were downregulated by the classic KD, whereas two genera, *Lachnospiraceae unclassified* and *Enterococcus*, were downregulated by the novel KD (Figure 5C; Supplementary Tables S1, S2). Hence, the classic KD downregulated more genera negatively correlated with plasma ketone levels than the novel KD and upregulated more genera positively correlated with plasma ketone levels, which is consistent with the higher ketosis induction observed with the classic KD than with the novel KD.^13^

### Correlations with plasma lipid levels

The individual dietary intake of fatty acids could not be reliably assessed due to food spilling in the cages. Therefore, we chose to correlate the plasma fatty acid levels to the microbiota changes, since plasma fatty acid levels are considered correlates of the amounts and sources of ingested fatty acids.^31^ Total free fatty acid plasma levels correlated positively with the abundance of one genus, *Escherichia Shigella*, and negatively with the abundances of *Bifidobacterium*, *Clostridium sensu stricto 1*, *Roseburia*, *Ruminococcus*, *Turicibacter*, and *GCA 900066575* (Figure 5B; Supplementary Tables S1, S2). The novel KD upregulated the abundances of *Bifidobacterium* and *Clostridium sensu stricto 1*. Meanwhile, the classic KD upregulated the abundance of *Escherichia Shigella* and downregulated the abundances of *Roseburia* and *Turicibacter* (Table 1). Thus, changes in genera abundances reflected the differences in plasma fatty acid levels between the two KDs.

**Figure 6.**
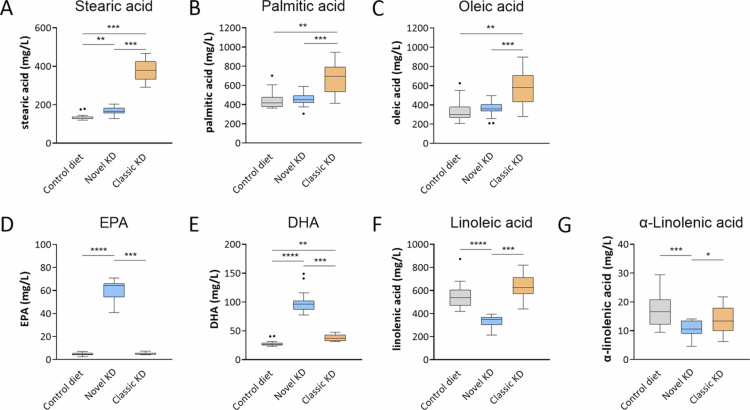
Plasma free fatty acid levels among kindled animals. Box and whisker plots of the plasma fatty acid concentrations, including (A) stearic acid, (B) palmitic acid, (C) oleic acid, (D) EPA, (E) DHA, (F) linoleic acid, and (G) α-linolenic acid. Fatty acid levels are expressed as mg/L, and dots represent outliers. Significance was assessed by pairwise Wilcoxon test *p < 0.05, **p < 0.01, ***p < 0.001, and ****p < 0.0001.

Stearic acid plasma concentrations, which were elevated by the classic KD compared to the control diet, with intermediate levels for the novel KD (Figure 6A), correlated positively with *Bacteroides*, *Tannerellaceae*, *Faecalibaculum*, and *Escherichia Shigella* abundances (Figure 5B; Supplementary Tables S1, S2). Of these, the classic KD upregulated *Bacteroides*, *Tannerellaceae*, and *Escherichia Shigella* more than the novel KD (Table 1). Meanwhile, palmitic acid and oleic acid plasma concentrations were elevated for the classic KD only (Figure 6B,C) and correlated positively with *Faecalibaculum* abundance (Figure 5B; Supplementary Tables S1, S2), which was increased by both KDs (Table 1). Furthermore, stearic acid, palmitic acid, and oleic acid plasma concentrations correlated negatively with *Clostridia UCG 014 ge*, *Ruminococcus*, *Roseburia*, *Turicibacter*, *Oscillospirales ge*, *Lachnospiraceae UCG 006*, *Lachnospiraceae unclassified*, *GCA 900066575*, and *Erysipelatoclostridium* (Figure 5B; Supplementary Tables S1, S2). Of these, the classic KD downregulated five genera, namely, *Clostridia UCG 014*, *Roseburia*, *Turicibacter*, *Erysipelatoclostridium*, *Lachnospiraceae UCG 006*, and *Oscillospirales ge*, whereas the novel KD downregulated two genera, *Oscillospirales ge* and *Lachnospiraceae unclassified* (Table 1). Hence, the classic KD altered a greater number of genera abundances correlated with greater stearic acid, palmitic acid, and oleic acid plasma concentrations than the novel KD.

Docosahexaenoic acid (DHA) and eicosapentaenoic acid (EPA) plasma concentrations correlated positively with *Bifidobacterium*, *Faecalibaculum*, *Clostridium sensu stricto 1*, *Muribaculaceae ge*, *Lactobacillus,* and *Lachnoclostridium* and negatively with *Akkermansia*, *Blautia*, *Erysipelatoclostridium, Staphylococcus, Escherichia Shigella*, *Oscillospirales ge*, *Peptococcaceae uncultured, Lachnospiraceae unclassified*, *Lachnospiraceae uncultured*, *Marvinbryantia, Enterococcus*, and *Faecalitalea* abundances (Figure 5B; Supplementary Tables S1, S2). The total omega-3 to omega-6 PUFA ratio showed similar correlations as EPA and DHA, only lacking a negative correlation with *Akkermansia* (Figure 5B; Supplementary Tables S1, S2). Of these genera, the novel KD upregulated the abundances of *Bifidobacterium*, *Faecalibaculum*, *Clostridium sensu stricto 1*, and *Muribaculaceae ge*, more than the classic KD (Table 1) and downregulated the abundances of *Oscillospirales ge*, *Lachnospiraceae unclassified*, and *Enterococcus*, while the classic KD downregulated the abundances of *Oscillospirales ge* and *Faecalitalea.* Therefore, supplementation of the novel KD with EPA and DHA, unlike the classic KD and control diets, led to higher EPA and DHA plasma concentrations (Figure 6D,E) correlated with specific changes in genera abundances.

Linoleic acid (LA) plasma concentrations correlated positively with *Akkermansia*, *Blautia*, and *Escherichia Shigella* abundances and negatively with *Bifidobacterium*, *Clostridium sensu stricto 1*, *Clostridia UCG 014 ge, Muribaculaceae ge*, *Ruminococcus*, *Roseburia*, *Turicibacter*, *Lachnoclostridium*, *Lachnospiraceae NK4A136 group*, *Lachnospiraceae UCG 006*, and *GCA 900066575* abundances. Among these genera, *Bifidobacterium*, *Clostridium sensu stricto 1*, *Muribaculaceae ge,* and *Lachnoclostridium* were upregulated by the novel KD compared with the control diet, suggesting that reducing the LA content in the novel KD and thus LA plasma levels compared to the classic KD and control diet (Figure 6F) helped to upregulate these genera. Meanwhile, the classic KD upregulated *Escherichia shigella* and downregulated *Roseburia* and *Turicibacter* abundances compared to the novel KD and control diet, suggesting that the higher LA content of the classic KD might steer these changes in genera abundances.

α-Linolenic acid (ALA) plasma levels correlated positively with *Enterococcus* and negatively with *Bacteroides*, *Tannerellaceae*, *Bifidobacterium*, *Muribaculaceae ge*, *Ruminococcus*, *Lachnoclostridium*, *GCA 900066575*, *Lachnospiraceae UCG 006, Colidextribacter,* and *Oscillospiraceae uncultured*. Since ALA plasma levels were lowered by the novel KD compared to the control diet (Figure 6G), their reduced availability could be linked to upregulation of *Bacteroides*, *Tannerellaceae*, *Bifidobacterium*, *Muribaculaceae ge*, and *Lachnoclostridium* compared to the control diet.

### Correlations with liver triglyceride levels

Liver triglyceride levels positively correlated with *Bacteroides*, *Tannerellaceae ge*, *Escherichia Shigella,* and *Blautia*, while negatively with *Turicibacter*, *Clostridia UCG 014 ge,* Lachnospiraceae *UCG 006*, *GCA 900066575*, *Roseburia,* and *Ruminococcus* (Figure 5C; Supplementary Tables S1, S2). They largely matched the correlations observed for stearic acid, palmitic acid, and oleic acid. Furthermore, they were aligned with increased abundances of *Bacteroides*, *Tannerellaceae ge*, *Escherichia Shigella* and decreased abundances of *Turicibacter*, *Lachnospiraceae UCG 006*, and *Roseburia* in the classic KD group (Table 1), which developed hepatic steatosis.^13^

### Correlations with seizure responses

Seizure responses (total number of Racine stage 1 s, latency to reach more severe stages 4, 5 and the fully kindled stage) in the rapid kindling rat model showed correlations with genera abundances (Figure 5D). Positive correlations were found for *Muribaculaceae ge* abundance for the number of Racine stage 1 s and latency to stage 4, *Clostridium sensu stricto 1* abundance for latency to stage 4 and latency to fully kindled, and *GCA 900066575* abundance for the number of Racine stage 1 s and latency to fully kindled. A negative correlation was found for *Staphylococcus* abundance for latency to stages 4 and 5 (Figure 5D; Supplementary Tables S1, S2). As *Clostridium sensu stricto 1* abundance was uniquely upregulated by the novel KD, while *Muribaculaceae ge* abundance was equally upregulated by both KDs, and *GCA 900066575* abundance was not altered compared to the control diet, and given that the novel KD had greater antiseizure efficacy,^13^ the data suggest *Clostridium sensu stricto 1* as a new genus associated with seizure protection.

## Discussion

A classic rodent-adapted 6:1 KD containing cellulose as the sole poorly-fermentable dietary fiber altered the gut microbiota composition and drastically reduced overall SCFA cecum levels, compared to a control diet also containing cellulose as its only dietary fiber. Meanwhile, a novel 1.9:1 KD with a lowered stearic acid content and supplemented with omega-3 PUFAs, and a dietary fiber blend composed of inulin, resistant starch, soy, and GOS, restored overall SCFA levels in the cecum and increased the butyrate-to-propionate molar ratio to control diet levels. While the fiber content was ~5 wt% for the classic KD and control diet, and ~11 wt% for the novel KD, the fiber content was ~0.006 g/kcal for the classic KD, ~0.013 g/kcal for the control diet, and ~0.02 g/kcal for the novel KD. This means that, per kcal ingested, the fiber content of the novel KD doubles and triples, respectively, the fiber content of the control diet and the classic KD, while the fiber content of the control diet roughly doubles the fiber content of the classic KD. Cellulose, a main component of plant cell walls, is rather weakly digested in the upper gastrointestinal tract and fermented to SCFAs and butyrate by gut microbes, especially in humans living in industrialized societies.^32^ Humans in nonindustrialized societies and rodents may retain some ability to ferment cellulose to SCFAs,^32^^,^^33^ Different SCFA cecum levels could therefore be related to different cellulose intakes between the diet groups. Hence, it cannot be excluded that – although cellulose is considered poorly fermentable – the amounts of ingested fibers contributed to differences in SCFA production and genera abundances in the gut.

Furthermore, the novel KD led to a distinct gut microbiota signature with unique increases in abundance of *Bifidobacterium* and *Clostridium sensu stricto I*. No differentially abundant genera were correlated with the rescue of SCFA production. Meanwhile, several genera were associated with decreased liver triglyceride accumulation in the novel KD. Furthermore, a few genera correlated with the seizure response in the novel KD. Since the novel KD reduced seizure severity progression and prevented hepatic steatosis development compared to the classic KD,^11^ it is likely that supplementing fermentable fibers and omega-3 PUFAs, while reducing stearic acid, altered the gut microbiota and contributed to delaying these outcomes. Finally, kindling did not influence the gut microbiome in this epileptogenesis model.

### Diet-induced gut microbiota signatures

Gut microbiota diversity, genera abundances, and microbiota-derived metabolites were affected primarily by diet, while kindling had no significant impact on these outcomes. Gut dysbiosis is a proposed risk factor for the development of epilepsy, and refractory epilepsy patients showed differences in gut microbiota composition, compared to healthy matched controls.^34^ Furthermore, several *in vivo* studies in epilepsy models have shown that changes in the gut microbiota could infer protection or increase susceptibility to seizure development.^35^ KD treatment for epilepsy patients with gut dysbiosis has antiseizure effects that are partially driven by the microbiome.^36^ Given that the gut‒brain axis is bidirectional in nature, it may be surprising that kindling did not significantly alter the composition of the gut microbiota. This may be attributed to the temporal difference between the last kindling session and cecum collection 11 d after the last kindling session.

At the phylum level, the classic KD increased the relative abundance of Bacteroidota at the expense of Firmicutes, which is in line with clinical observations to date.^4^ A low Bacteroidota-to-Firmicutes ratio is a proposed marker of dysbiosis in the obesity-associated microbiome,^37^ which may be corrected with KD treatment. While the novel KD also increased the abundance of Bacteroidota, compared to the control diet, no reduction in Firmicutes was observed. Whether this difference in the Bacteroidota-to-Firmicutes ratio reflects differences in gut health remains to be clarified.

The novel KD lowered the alpha diversity in the Chao1 index when the evenness of the genus distribution was not considered for analysis, whereas it increased the alpha diversity when evenness was weighted in the Simpson index, compared to the control diet. Meanwhile, the classic KD did not alter alpha diversity, compared to the control diet. The sparse clinical evidence available in epilepsy target populations shows ambivalent results of KDs on alpha diversity. Two studies reported unchanged alpha diversity,^38^^,^^39^ and one study reported reduced alpha diversity.^40^

The three diet groups were also separated in beta diversity by Bray–Curtis PCA, with equal homogeneity between groups. Both KDs appeared to be less different from each other than from the control diet in terms of beta diversity by Bray–Curtis PCA. This suggests that the fiber blend and/or presence of specific nutrients such as omega-3 fatty acids or saturated long-chain fatty acids was sufficient to shift the beta diversity of the novel KD away from the classic KD.

Both KDs showed overlapping as well as unique changes in genera abundances. In line with previous studies, *the abundance of Bacteroides*, *Tannerellaceae ge*, *Muribaculaceae ge*, and *Faecalibaculum* were elevated predominantly by the classic KD, while *Akkermansia* abundance was not altered compared to the control diet. *Akkermansia* abundance was lower in the novel KD compared to the classic KD. We found that *Akkermansia* was inversely correlated with plasma EPA and DHA levels, suggesting that the greatly increased omega-3 fatty acid content in the novel KD group blocked the expansion of this genus. A previous report found that *Akkermansia* was upregulated with classic KD and might be related to the absence of omega-3 fatty acid supplementation.^6^

*The abundances of Bifidobacterium* and *Clostridium sensu stricto I* were upregulated only by the novel KD. Since the novel KD was less ketogenic than the classic KD,^13^ this finding might be related in part to the reported reduction in Actinobacteriota (including *Bifidobacterium*),^4^ and the selective inhibition of *Bifidobacterium* growth by β-hydroxybutyrate.^41^ Probably, a larger contributing reason is the dependence of *Bifidobacterium* for growth on fermentable fibers, such as inulin^42^^,^^43^ contained in the novel KD but not in the classic KD or control diet. Interestingly, *Bifidobacterium* and *Clostridium sensu stricto I* abundances were positively correlated with DHA and EPA plasma levels and the omega-3 to omega-6 PUFA plasma level ratio. The omega-3 to omega-6 plasma level ratio was further positively correlated with *Fecalibaculum* and *Muribaculaceae ge* abundances, which were increased by the novel KD and to a lesser degree by the classic KD relative to the control diet. Conversely, *Bifidobacterium*, *Clostridium sensu stricto I*, and *Muribaculaceae ge* abundances were negatively correlated with LA plasma concentrations, suggesting that LA blocked the growth of *Bifidobacterium* and *Muribaculaceae ge*. Since the novel KD contained DHA and EPA in exchange for LA,^13^ the resulting increase in the omega-3 to omega-6 PUFA weight ratio could have expanded *Bifidobacterium*, *Clostridium sensu stricto I, Faecalibaculum,* and *Muribaculaceae ge*. Supporting this notion, a clinical trial in healthy volunteers^44^ and an animal study in genetically obese (*LepR*^db/db^) mice^45^ showed that DHA and EPA increase *Bifidobacterium* abundance in the gut.

Furthermore, the abundances of many genera (including *Bacteroides*) correlated positively or negatively with stearic acid plasma levels, and to a lesser degree with palmitic acid and oleic acid plasma concentrations. A link between saturated fatty acids and *Bacteroides* abundance is supported by human *in vitro* fermentation assays showing that palmitic acid stimulated the growth of *Bacteroides fragilis* and *Bacteroides thetaiotaomicron*.^46^ Furthermore, resistant starch present only in the novel KD could have limited *Bacteroides* growth, as shown for *Bacteroides stercoris* in the human gut.^47^

### Diet effects on SCFA cecum levels

In the present study, the classic KD dramatically reduced SCFA and ammonia levels in the cecum. This finding has clinical relevance, as similar reductions in fecal SCFAs were observed in a one-month study with epileptic patients on a KD with 42% reduced fiber intake, compared to preintervention and without prebiotic or probiotic supplementation.^11^ This study reported 55% reduction in overall SCFA level, and 64% in acetate, 33% in propionate, and 20% in butyrate. This clinical observation of reduced SCFA is supported by several studies using low-carbohydrate diets intended for weight loss, where a similar disproportional stronger reduction in butyrate production was observed.^48-50^ Conversely, a higher intake of cruciferous and leafy vegetables, berries and nuts (all containing high amounts of dietary fiber) with a KD in healthy adults led to higher butyrate levels compared to the control group on a normal diet with lower dietary fiber intake.^51^ Similarly, high-fat diets (HFDs), containing less fat but more carbohydrates and proteins than a KD, can also reduce the total SCFA stool content in rodents by foremost impacting the acetate fraction.^52^ The inclusion of dietary fibers from basil seed flour or oat flour was found to restore the total SCFA content by partly rescuing the acetate stool content and upregulating the propionic acid and butyrate stool contents. The inclusion of basil seed and oat flour fibers further decreased HFD-induced liver steatosis, suggesting that SCFAs convey this health benefit.^52^

SCFA induce physiological responses by binding to G protein-coupled receptors on cell plasma membranes. This may drive β-oxidation and ketone production in liver cells.^53^ This could have prevented fatty liver development in the novel KD group.^13^ Conversely, the loss of SCFA receptor activation could have favored lipid accumulation in hepatocytes with a classic KD. Reduced butyrate levels and a decreased butyrate-to-propionate ratio could render hepatic ketogenesis less efficacious, since butyrate is a ketogenic enhancer,^12^ meaning that more fat is needed to sustain ketone production. Furthermore, decreased butyrate production could adversely influence gut and metabolic health.^8^

Besides qualitative and quantitative differences in fiber content, varying digestible carbohydrate and protein contents could be related to SCFA production, as partly undigested carbohydrates in the small intestine may leak into the colon for fermentation to SCFAs,^2^ contributing substantially to SCFA cecum levels in the control diet group. Undigested proteins can also be fermented to SCFAs^54^, mostly acetate (~50 wt%), and less so butyrate and propionate (<20 wt%). Therefore, the low carbohydrate content (control diet vs classic KD: 181.5 vs 5.7 mg/kcal) and low protein content of the classic KD (control diet vs classic KD: 36.2 vs 10.9 mg/kcal) could have further limited the substrate available for fermentation to SCFAs. The cecum levels of ammonia, isobutyrate, and isovalerate, which are normally produced by the gut through proteolytic fermentation, were highest in the control diet group and likely reflect differences in dietary protein content.

### Genera abundances correlating with SCFA cecum level

Four genera were positively correlated with cecum butyrate and acetate levels and increased in abundance in the novel KD compared to the classic KD: *Turicibacter, Clostridia UCG 014 ge, Lachnospiraceae UCG 006,* and *Roseburia*. These genera are likely candidates for the restoration of cecum acetate and butyrate levels in the novel KD group. Surprisingly, *Bifidobacterium* did not positively correlate with SCFA production. *Bifidobacterium* has many acetate-producing members,^55^ and it may still be involved in the restoration of butyrate production, because this acetate can cross-feed butyrate-producing genera^56^ without altering *Bifidobacterium* abundance. The classic KD altered far more genera in a manner correlated with lower SCFA cecum levels than the novel KD, which may explain the dramatic loss of SCFA production in the classic KD group.

### Genera abundances correlating with dietary lipid content and fatty liver development

The classic KD altered the microbiota composition in a manner more closely correlated with liver triglyceride accumulation than the novel KD. The classic KD upregulated by far more genera abundances positively correlating with the liver triglyceride content, and downregulated more genera abundances correlating negatively with the liver triglyceride content than the novel KD. Furthermore, these genera abundances correlated mostly with the stearic acid plasma concentrations and weakly with the palmitic acid and oleic acid plasma concentrations, which were all elevated by the classic KD but not the novel KD. In contrast, neither the EPA or DHA plasma concentrations nor the omega-3 to omega-6 PUFA plasma concentration ratio correlated with these genera. DHA and EPA plasma concentrations and the omega-3 to omega-6 PUFA plasma concentration ratio correlated positively with *Bifidobacterium* and *Clostridium sensu stricto I* abundances, suggesting that DHA and EPA are probiotics for both genera, but their abundances were not correlated with liver triglyceride content. The LA plasma concentration correlated positively with *Escherichia Shigella* abundance, and negatively with *Roseburia* and *Turicibacter* abundances, hinting towards LA driving microbiota changes favoring hepatic triglyceride accumulation. Correlations to genera abundances related to fatty acid content in the plasma and not in the cecum. Hence, plasma fatty acid levels might have been influenced by the gut microbiota or vice versa. Providing DHA, lowering LA, and/or increasing the omega-3 to omega-6 PUFA weight ratio in the diet may also directly contribute to reducing hepatic steatosis and inflammation. Furthermore, the provision of DHA could improve mitochondrial dysfunction in the context of hepatic steatosis and thereby help the upregulation of β-oxidation and the clearance of fatty acids in the liver.^57^

*Bacteroides*, which was more abundant in the classic KD group than in the novel KD group, included strains linked to fatty liver development. For instance, *Bacteroides* stercoris has been shown to enhance hepatic lipid accumulation through fecal microbiota transplantation and monocolonization.^44^

Although *Bifidobacterium* abundance does not correlate with liver triglyceride content, it might still prevent liver steatosis because *Bifidobacterium* abundance is negatively correlated with free fatty acid plasma concentrations, which remain as low as the control diet levels, whereas they are elevated by the classic KD.^13^ Supporting this view, previous studies identified *B. breve*,^58^ *B. longum*,^58^ and *B. lactis V9* ^59^ as probiotics to attenuate steatosis and inflammatory biomarkers in high-fat diet-induced nonalcoholic fatty liver disease (NAFLD) models. This was associated with a heightened production of SCFAs in the gut and an improved gut barrier. Moreover, *B. adolescentis* reduced hepatic steatosis by sensitizing the fibroblast growth factor 21 pathway, which mediates ketone production in liver cells.^60^ Finally, rescuing SCFA production with *Bifidobacterium* may help not only against hepatic steatosis but also against the consequences thereof, as *B. pseudolongum* protected against NAFLD-associated hepatocellular carcinoma by increasing gut acetate production.^61^

Less is known about the impact of *Clostridium sensu stricto I* on liver metabolic health. One study noted that downregulation of fecal *Clostridium sensu stricto I* abundance in humans with NAFLD was associated with a more severe degree of liver steatosis and liver fibrosis.^62^ Another study indicated that *Clostridium sensu stricto* is significantly associated with protection against hepatocellular carcinoma development in cirrhosis patients.^63^ Therefore, the ketosis boosting nutrients, altered macronutrient composition, as well as reduced the abundance of *Bacteroides* together with the unique expansion of protective genera may have inferred protection from fatty liver development in novel KD-treated animals.

### Genera abundances correlating with seizure response

The novel KD and nutrients contained therein, most likely have a direct behavioral seizure-reducing effect. The butyrogenic fiber blend was added to stimulate hepatic ketogenesis, but it may have stimulated the expansion of the microbiota with the production of metabolites with beneficial effects on epileptogenic processes. The number of Racine stage 1 seizures and latency to reach stage 4 seizures, or the fully kindled state in the rapid kindling model were all increased by the novel KD compared to the classic KD and the control diet,^13^ reflecting the superior antiseizure efficacy of the novel KD. *Muribaculaceae ge*, *Clostridium sensu stricto I*, and *GCA 900066575* abundances were positively correlated with these seizure scores. Among these genera, *Clostridium sensu stricto I* and *Muribaculaceae ge* abundances were upregulated by the novel KD, whereas only the latter was upregulated by the classic KD, and *GCA 900066575* abundance was not altered by either KD. Therefore, *Clostridium sensu stricto I* is an interesting candidate for further exploration of the improved antiseizure efficacy of the novel KD over the classic KD.^13^ Besides, one study, where lower *Clostridium sensu stricto 1* abundance was associated with focal epilepsy development in humans,^64^ its role in epilepsy development is elusive. Similarly, the role of *Muribaculaceae* in the development of epilepsy warrants further investigation, as the depletion of *Muribaculaceae* strains has been shown to increase the susceptibility of rats to posttraumatic epilepsy.^62^ In contrast, more evidence for the antiseizure activity of *Bifidobacterium* exists. It was shown to produce the inhibitory neurotransmitter γ-aminobutyric acid (GABA),^65^ and its abundance was lower in drug-resistant epilepsy patients compared to those, who responded well to antiseizure medications.^66^ Bifidobacterium was effective as an antiseizure probiotic in a chronic epilepsy rat model,^67^ as well as a synbiotic in combination with inulin, further increasing the antiepileptogenic and antineuroinflammatory efficacy in this model.^67^ In the present study, *Bifidobacterium* did not correlate with behavioral seizure severity during kindling. However, these are electrically induced seizures, and therefore, the contribution of *Bifidobacterium* and to suppress recurrent spontaneous seizures cannot be excluded. Furthermore, *Bifidobacterium* could cross-feed seizure-protecting but not differentially abundant genera. Assessing the significance between genera abundances and behavioral seizure severity is challenging given the discrete valuation of the seizure scores on a scale from 1 to 5 and fully kindled.

## Conclusions

This work describes a novel ketogenic diet that corrects SCFA levels in the gut and elicits gut microbiota changes that correlate with seizure scores and liver triglyceride content.

Further studies are needed to evidence that the change in gut microbiome composition contributes to the therapeutic efficacy of the novel KD. To demonstrate a cause‒effect relationship, a fecal microbiota transplantation experiment would be the gold standard. In addition, tissue metabolomics can identify gut microbiota-derived metabolites and their impact on host metabolism.^68^ These specific metabolites could be further evaluated in high throughput cell-based assays for their effects on hepatic lipid metabolism and neuroexcitability, further supporting a causal relationship between gut microbiota composition changes and therapeutic outcomes. In addition, measuring metabolites derived from the intestinal microbiota will improve the understanding of the broader relevance of the results, since animal facilities differ with respect to intestinal microbiota composition. In this context, metabolomics can help differentiate bacterial metabolites originating from the facility's baseline microbiota from those specifically elicited or superimposed by different ketogenic diets. This is relevant to determine the role of the intestinal microbiota composition in mediating the effects of the different ketogenic diets.

To define the associations at species and strain levels and identify potential probiotics, shotgun metagenomic sequencing of the cecum could be performed. Highly correlated strains/species could be further assessed in animal and human clinical trials for their efficacy in conjunction with prebiotic fibers.

Diet is recognized as important for the prevention and treatment of metabolic dysfunction-associated fatty liver disease (MAFLD) or metabolic dysfunction-associated steatotic liver disease (MASLD).^69^ The findings of this work can be further elaborated on to develop probiotics and synbiotics to support the dietary treatment of refractory epilepsy and MAFLD/MASLD. Pro- and synbiotics improving the antiseizure efficacy of the KD would allow epilepsy patients to ingest a less restrictive KD that is less fat-containing and less ketone-dependent but equally effective KD and to reduce the risk for developing MAFLD/MASLD.

## Supplementary Material

Supplementary material Figure S1. Hierarchical clustering including the nonkindled groups.

Supplementary material Figure S2. Microbiota metabolites and cecum pH among all groups.

Supplementary material Table S1. Correlation coefficients of correlations with phenotypic outcomes.

Supplementary material Table S2. Adjusted *p*-values of correlations with phenotypic outcomes.

Supplementary material Supplementary material

## Data Availability

SCFA, metabolite and pH data are available from the corresponding author upon reasonable request.

## Data deposition

The 16S rRNA sequencing reads, assembled reads, counts and differential abundance calculations can be publicly accessed in the European Nucleotide Archive (ENA) with project number PRJEB78951.
