# Supplementary figures and images for: A novel ketogenic diet that reduces seizures and prevents liver steatosis leads to related gut microbiome changes and restores cecal short-chain fatty acid levels in the rapid kindling rat model of epileptogenesis
Source: Gut Microbes Rep. 2025 Oct 9;2(1):2567677. doi: 10.1080/29933935.2025.2567677 (PMC12899332; doi:10.1080/29933935.2025.2567677)

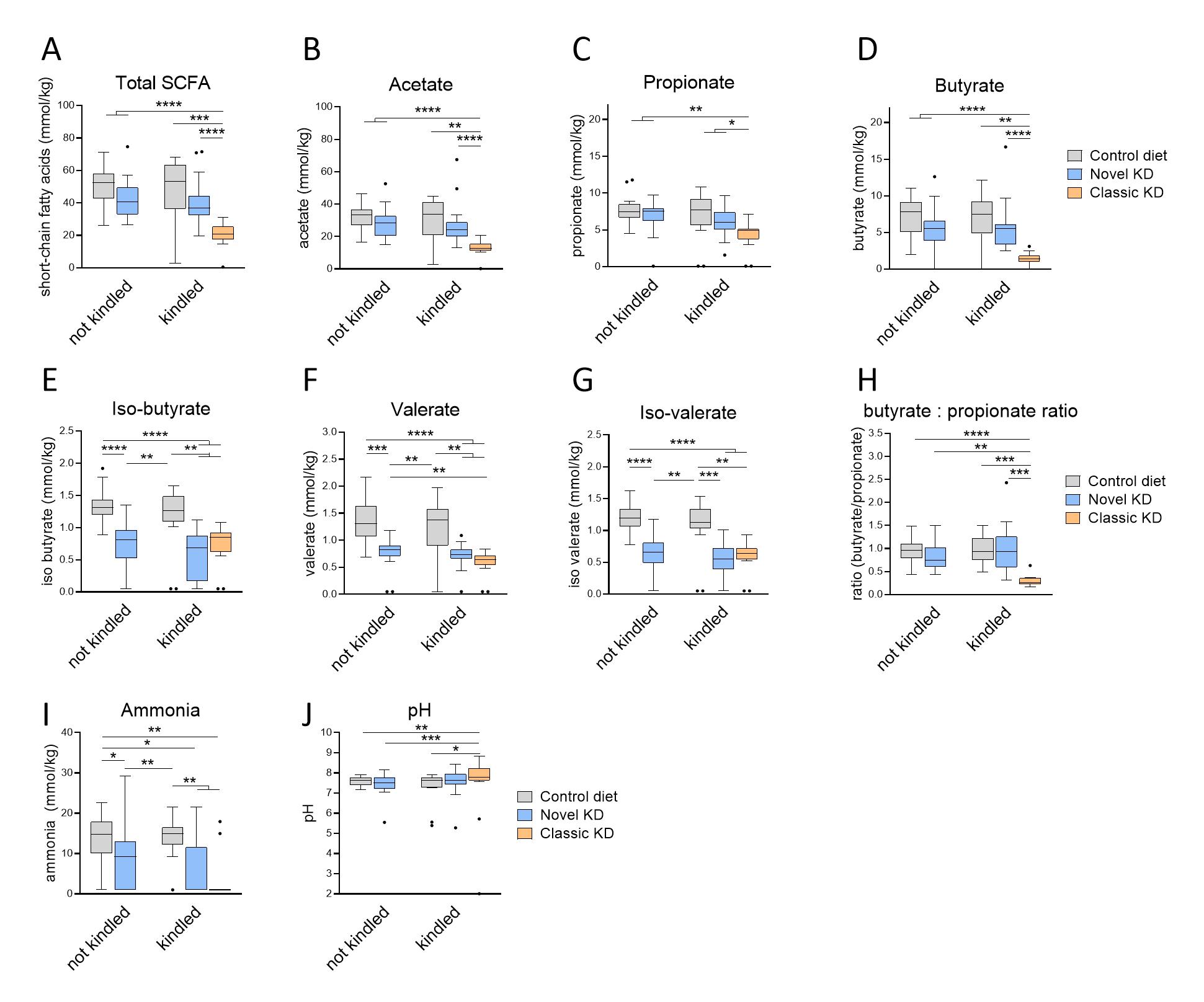

Supplement: Supplementary material — Figure S1. Hierarchical clustering including the nonkindled groups. [file KGMR_A_2567677_SM3051.jpg]
