# Supplementary material for: A novel ketogenic diet that reduces seizures and prevents liver steatosis leads to related gut microbiome changes and restores cecal short-chain fatty acid levels in the rapid kindling rat model of epileptogenesis
Source: Gut Microbes Rep. 2025 Oct 9;2(1):2567677. doi: 10.1080/29933935.2025.2567677 (PMC12899332; doi:10.1080/29933935.2025.2567677)
Supplement: Supplementary material — Table S1. Correlation coefficients of correlations with phenotypic outcomes. [file KGMR_A_2567677_SM3054.docx]

|  | **Acetic acid** | **Propionic acid** | **Iso-butyrate** | **Butyrate** | **Iso-valerate** | **Valerate** | **butyrate : propionate ratio** | **Myristic acid** | **Palmitic acid** | **Stearic acid** | **Oleic acid** | **Linoleic acid** | **alpha Linolenic acid** | **EPA** | **DHA** | **Liver triglycerides** | **omega-3 to-6 ratio** | **Body weight** | **Ketones** | **Liver triglyceride** | **Kindling Number Stage1** | **Kindling Latency to Stage4** | **Kindling Latency to Stage5** | **Kindling Latency to Fully Kindled** |
| --- | --- | --- | --- | --- | --- | --- | --- | --- | --- | --- | --- | --- | --- | --- | --- | --- | --- | --- | --- | --- | --- | --- | --- | --- |
| **Bifidobacterium** | 0.04 | -0.15 | -0.50 | 0.02 | -0.47 | -0.31 | 0.24 | 0.21 | -0.12 | 0.07 | -0.06 | -0.65 | -0.34 | 0.65 | 0.72 | -0.45 | 0.73 | -0.05 | 0.11 | -0.21 | 0.28 | 0.29 | 0.27 | 0.25 |
| **Bacteroides** | -0.36 | -0.23 | -0.22 | -0.29 | -0.22 | -0.25 | -0.41 | 0.05 | 0.28 | 0.58 | 0.29 | -0.06 | -0.35 | 0.12 | 0.31 | 0.14 | 0.25 | -0.13 | 0.53 | 0.42 | 0.02 | 0.01 | 0.01 | -0.13 |
| **Muribaculaceae ge** | -0.04 | -0.08 | -0.27 | 0.13 | -0.32 | -0.06 | 0.05 | 0.01 | -0.03 | 0.23 | 0.00 | -0.48 | -0.45 | 0.40 | 0.52 | -0.30 | 0.51 | 0.01 | 0.23 | 0.00 | 0.30 | 0.33 | 0.29 | 0.14 |
| **Alistipes** | -0.29 | -0.22 | -0.08 | -0.15 | -0.10 | -0.16 | -0.25 | -0.10 | 0.12 | 0.28 | 0.11 | -0.05 | -0.31 | 0.00 | 0.14 | 0.03 | 0.07 | -0.15 | 0.22 | 0.14 | 0.05 | 0.08 | 0.10 | -0.14 |
| **Tannerellaceae ge** | -0.38 | -0.20 | -0.08 | -0.30 | -0.09 | -0.21 | -0.47 | -0.05 | 0.25 | 0.54 | 0.26 | 0.02 | -0.35 | -0.02 | 0.16 | 0.16 | 0.12 | -0.19 | 0.52 | 0.39 | -0.01 | -0.03 | 0.01 | -0.15 |
| **Erysipelatoclostridium** | 0.42 | 0.69 | 0.74 | 0.41 | 0.73 | 0.64 | -0.03 | -0.39 | -0.51 | -0.51 | -0.51 | -0.13 | -0.12 | -0.36 | -0.45 | -0.30 | -0.35 | 0.01 | -0.46 | -0.16 | -0.11 | -0.19 | -0.15 | -0.03 |
| **Faecalibaculum** | -0.26 | -0.38 | -0.65 | -0.30 | -0.62 | -0.58 | -0.04 | 0.51 | 0.34 | 0.49 | 0.41 | -0.30 | -0.14 | 0.58 | 0.72 | 0.00 | 0.68 | -0.10 | 0.41 | 0.21 | 0.25 | 0.31 | 0.25 | 0.11 |
| **Faecalitalea** | 0.32 | 0.34 | 0.39 | 0.37 | 0.36 | 0.46 | 0.23 | -0.05 | -0.20 | -0.34 | -0.22 | 0.09 | 0.17 | -0.30 | -0.42 | -0.09 | -0.35 | 0.39 | -0.44 | -0.12 | -0.21 | -0.18 | -0.21 | -0.02 |
| **Turicibacter** | 0.44 | 0.21 | 0.04 | 0.42 | 0.00 | 0.24 | 0.54 | -0.02 | -0.41 | -0.57 | -0.40 | -0.42 | 0.03 | 0.20 | 0.11 | -0.48 | 0.12 | 0.24 | -0.52 | -0.50 | 0.14 | 0.21 | 0.14 | 0.31 |
| **Enterococcus** | 0.18 | 0.13 | 0.39 | 0.18 | 0.40 | 0.31 | 0.24 | 0.02 | -0.11 | -0.41 | -0.15 | 0.30 | 0.43 | -0.33 | -0.47 | 0.08 | -0.46 | 0.07 | -0.39 | -0.15 | -0.20 | -0.21 | -0.27 | -0.06 |
| **Lactobacillus** | -0.34 | -0.45 | -0.63 | -0.38 | -0.61 | -0.58 | -0.01 | 0.35 | 0.22 | 0.30 | 0.28 | -0.21 | -0.09 | 0.52 | 0.60 | -0.04 | 0.50 | -0.13 | 0.28 | 0.04 | 0.12 | 0.19 | 0.22 | 0.06 |
| **Staphylococcus** | 0.29 | 0.33 | 0.44 | 0.37 | 0.48 | 0.48 | 0.26 | -0.30 | -0.32 | -0.52 | -0.37 | 0.26 | 0.28 | -0.42 | -0.67 | -0.06 | -0.61 | 0.04 | -0.50 | -0.25 | -0.18 | -0.31 | -0.32 | -0.18 |
| **Clostridia UCG 014 ge** | 0.65 | 0.53 | 0.34 | 0.67 | 0.38 | 0.52 | 0.52 | -0.15 | -0.49 | -0.59 | -0.46 | -0.33 | 0.02 | 0.02 | -0.16 | -0.38 | -0.04 | 0.28 | -0.56 | -0.42 | 0.10 | 0.09 | 0.09 | 0.14 |
| **Clostridium sensu stricto 1** | -0.06 | -0.21 | -0.52 | -0.06 | -0.54 | -0.34 | 0.20 | 0.14 | -0.08 | 0.01 | -0.03 | -0.48 | -0.21 | 0.56 | 0.61 | -0.32 | 0.56 | 0.00 | 0.05 | -0.08 | 0.28 | 0.33 | 0.27 | 0.30 |
| **Blautia** | 0.07 | 0.22 | 0.37 | 0.02 | 0.34 | 0.22 | -0.19 | -0.04 | 0.03 | 0.03 | 0.06 | 0.45 | 0.20 | -0.51 | -0.58 | 0.28 | -0.59 | 0.03 | -0.09 | 0.30 | -0.27 | -0.25 | -0.18 | -0.12 |
| **GCA 900066575** | 0.20 | 0.39 | 0.23 | 0.33 | 0.19 | 0.29 | 0.02 | -0.53 | -0.53 | -0.34 | -0.52 | -0.51 | -0.52 | 0.22 | 0.20 | -0.51 | 0.28 | -0.16 | -0.05 | -0.41 | 0.32 | 0.19 | 0.29 | 0.32 |
| **Lachnoclostridium** | 0.00 | 0.03 | -0.13 | 0.14 | -0.09 | 0.05 | -0.04 | -0.22 | -0.14 | 0.11 | -0.20 | -0.45 | -0.48 | 0.21 | 0.32 | -0.33 | 0.34 | 0.05 | 0.14 | -0.01 | 0.23 | 0.19 | 0.13 | 0.04 |
| **Lachnospiraceae NK4A136 group** | 0.30 | 0.28 | 0.09 | 0.45 | 0.10 | 0.38 | 0.26 | -0.10 | -0.22 | -0.18 | -0.23 | -0.38 | -0.16 | 0.23 | 0.20 | -0.32 | 0.30 | 0.18 | -0.21 | -0.22 | 0.27 | 0.20 | 0.17 | 0.04 |
| **Lachnospiraceae UCG 006** | 0.55 | 0.58 | 0.30 | 0.66 | 0.25 | 0.52 | 0.36 | -0.40 | -0.64 | -0.62 | -0.68 | -0.65 | -0.34 | 0.27 | 0.16 | -0.71 | 0.28 | 0.08 | -0.45 | -0.61 | 0.29 | 0.24 | 0.28 | 0.24 |
| **Lachnospiraceae unclassified** | 0.41 | 0.41 | 0.57 | 0.48 | 0.57 | 0.58 | 0.17 | -0.28 | -0.34 | -0.36 | -0.36 | 0.02 | 0.01 | -0.37 | -0.51 | -0.10 | -0.48 | 0.05 | -0.47 | -0.12 | -0.19 | -0.16 | -0.12 | -0.14 |
| **Marvinbryantia** | 0.21 | 0.35 | 0.47 | 0.22 | 0.41 | 0.46 | -0.10 | -0.37 | -0.26 | -0.18 | -0.26 | 0.11 | -0.11 | -0.41 | -0.49 | -0.06 | -0.44 | -0.09 | -0.09 | 0.10 | -0.06 | -0.10 | -0.05 | -0.05 |
| **Roseburia** | 0.34 | 0.36 | 0.36 | 0.46 | 0.42 | 0.44 | 0.24 | -0.25 | -0.53 | -0.46 | -0.50 | -0.44 | -0.30 | 0.03 | 0.02 | -0.50 | 0.05 | -0.03 | -0.44 | -0.39 | 0.01 | 0.00 | 0.06 | 0.00 |
| **Lachnospiraceae uncultured** | 0.09 | 0.18 | 0.49 | 0.14 | 0.44 | 0.35 | -0.15 | -0.39 | -0.19 | -0.11 | -0.25 | 0.07 | -0.22 | -0.55 | -0.44 | -0.04 | -0.42 | 0.03 | -0.10 | 0.05 | -0.03 | -0.01 | -0.08 | -0.10 |
| **Colidextribacter** | -0.26 | -0.19 | 0.06 | -0.16 | 0.04 | -0.07 | -0.35 | -0.19 | 0.10 | 0.31 | 0.08 | -0.01 | -0.34 | -0.11 | 0.04 | 0.08 | -0.02 | -0.17 | 0.27 | 0.23 | -0.05 | -0.02 | 0.02 | -0.23 |
| **Oscillospiraceae uncultured** | -0.14 | -0.08 | 0.08 | 0.00 | 0.03 | 0.06 | -0.16 | -0.30 | -0.03 | 0.16 | -0.06 | -0.13 | -0.40 | -0.01 | 0.09 | -0.07 | 0.07 | -0.03 | 0.17 | 0.05 | 0.21 | 0.21 | 0.25 | 0.05 |
| **Oscillospirales ge** | 0.38 | 0.53 | 0.76 | 0.42 | 0.81 | 0.63 | 0.05 | -0.26 | -0.34 | -0.50 | -0.36 | 0.13 | 0.16 | -0.50 | -0.63 | -0.06 | -0.55 | 0.03 | -0.56 | -0.22 | -0.23 | -0.28 | -0.28 | -0.21 |
| **Ruminococcus** | 0.26 | 0.18 | 0.08 | 0.42 | 0.11 | 0.32 | 0.30 | -0.41 | -0.53 | -0.40 | -0.54 | -0.44 | -0.32 | -0.05 | -0.08 | -0.49 | 0.07 | 0.14 | -0.11 | -0.35 | 0.15 | 0.08 | 0.08 | 0.17 |
| **Peptococcaceae uncultured** | 0.27 | 0.46 | 0.64 | 0.30 | 0.63 | 0.54 | -0.14 | -0.35 | -0.25 | -0.31 | -0.28 | 0.18 | 0.06 | -0.49 | -0.54 | 0.01 | -0.46 | -0.06 | -0.28 | -0.12 | -0.08 | -0.17 | -0.07 | -0.04 |
| **Romboutsia** | 0.05 | -0.06 | -0.23 | 0.00 | -0.22 | -0.10 | 0.18 | 0.18 | -0.01 | -0.10 | -0.04 | -0.12 | 0.12 | 0.11 | 0.15 | -0.10 | 0.14 | 0.13 | -0.16 | -0.13 | -0.04 | -0.03 | -0.09 | 0.06 |
| **Escherichia Shigella** | -0.28 | -0.03 | 0.18 | -0.25 | 0.22 | -0.03 | -0.34 | 0.08 | 0.30 | 0.32 | 0.23 | 0.47 | 0.25 | -0.45 | -0.33 | 0.45 | -0.33 | -0.05 | 0.12 | 0.42 | -0.19 | -0.23 | -0.24 | -0.32 |
| **Akkermansia** | -0.10 | 0.30 | 0.50 | -0.11 | 0.50 | 0.20 | -0.52 | -0.15 | 0.12 | 0.11 | 0.09 | 0.33 | 0.03 | -0.39 | -0.36 | 0.26 | -0.29 | -0.19 | 0.16 | 0.21 | -0.16 | -0.27 | -0.26 | -0.26 |

**Table S1 correlation coefficients of correlations with phenotypic outcomes**
