# Supplementary material for: A novel ketogenic diet that reduces seizures and prevents liver steatosis leads to related gut microbiome changes and restores cecal short-chain fatty acid levels in the rapid kindling rat model of epileptogenesis
Source: Gut Microbes Rep. 2025 Oct 9;2(1):2567677. doi: 10.1080/29933935.2025.2567677 (PMC12899332; doi:10.1080/29933935.2025.2567677)
Supplement: Supplementary material — Table S2. Adjusted p-values of correlations with phenotypic outcomes. [file KGMR_A_2567677_SM3056.docx]

|  | **Acetic acid** | **Propionic acid** | **Iso-butyrate** | **Butyrate** | **Iso-valerate** | **Valerate** | **butyrate : propionate ratio** | **Myristic acid** | **Palmitic acid** | **Stearic acid** | **Oleic acid** | **Linoleic acid** | **alpha Linolenic acid** | **EPA** | **DHA** | **Liver triglycerides** | **omega-3 to-6 ratio** | **Body weight** | **Ketones** | **Liver triglyceride** | **Kindling Number Stage1** | **Kindling Latency to Stage4** | **Kindling Latency to Stage5** | **Kindling Latency to Fully Kindled** |
| --- | --- | --- | --- | --- | --- | --- | --- | --- | --- | --- | --- | --- | --- | --- | --- | --- | --- | --- | --- | --- | --- | --- | --- | --- |
| **Bifidobacterium** | 0.8119 | 0.3277 | 0.0004 | 0.8852 | 0.0011 | 0.0353 | 0.1077 | 0.1887 | 0.4534 | 0.6558 | 0.7037 | 0.0000 | 0.0296 | 0.0000 | 0.0000 | 0.0030 | 0.0000 | 0.7635 | 0.4958 | 0.1639 | 0.0625 | 0.0539 | 0.0757 | 0.0914 |
| **Bacteroides** | 0.0138 | 0.1229 | 0.1426 | 0.0549 | 0.1425 | 0.1039 | 0.0054 | 0.7633 | 0.0683 | 0.0001 | 0.0615 | 0.7216 | 0.0239 | 0.4349 | 0.0435 | 0.3695 | 0.1104 | 0.4036 | 0.0002 | 0.0040 | 0.8973 | 0.9232 | 0.9499 | 0.4085 |
| **Muribaculaceae ge** | 0.8105 | 0.5844 | 0.0720 | 0.4101 | 0.0319 | 0.6999 | 0.7374 | 0.9655 | 0.8365 | 0.1468 | 0.9801 | 0.0014 | 0.0030 | 0.0087 | 0.0005 | 0.0505 | 0.0005 | 0.9524 | 0.1404 | 0.9986 | 0.0476 | 0.0280 | 0.0510 | 0.3421 |
| **Alistipes** | 0.0547 | 0.1541 | 0.5891 | 0.3390 | 0.4943 | 0.3054 | 0.0934 | 0.5297 | 0.4671 | 0.0713 | 0.4735 | 0.7734 | 0.0446 | 0.9992 | 0.3690 | 0.8605 | 0.6646 | 0.3271 | 0.1497 | 0.3648 | 0.7284 | 0.5788 | 0.5078 | 0.3717 |
| **Tannerellaceae ge** | 0.0090 | 0.1828 | 0.6065 | 0.0472 | 0.5734 | 0.1618 | 0.0012 | 0.7377 | 0.1068 | 0.0002 | 0.0987 | 0.8934 | 0.0232 | 0.8773 | 0.3104 | 0.3138 | 0.4664 | 0.1994 | 0.0003 | 0.0086 | 0.9287 | 0.8660 | 0.9643 | 0.3147 |
| **Erysipelatoclostridium** | 0.0043 | 0.0000 | 0.0000 | 0.0047 | 0.0000 | 0.0000 | 0.8555 | 0.0116 | 0.0006 | 0.0006 | 0.0005 | 0.4271 | 0.4646 | 0.0208 | 0.0026 | 0.0522 | 0.0226 | 0.9558 | 0.0018 | 0.3058 | 0.4788 | 0.2206 | 0.3119 | 0.8307 |
| **Faecalibaculum** | 0.0805 | 0.0103 | 0.0000 | 0.0486 | 0.0000 | 0.0000 | 0.7938 | 0.0006 | 0.0299 | 0.0010 | 0.0068 | 0.0577 | 0.3929 | 0.0001 | 0.0000 | 0.9943 | 0.0000 | 0.5002 | 0.0059 | 0.1588 | 0.1044 | 0.0391 | 0.1042 | 0.4786 |
| **Faecalitalea** | 0.0349 | 0.0235 | 0.0086 | 0.0114 | 0.0142 | 0.0015 | 0.1361 | 0.7503 | 0.2017 | 0.0299 | 0.1621 | 0.5565 | 0.2768 | 0.0519 | 0.0060 | 0.5556 | 0.0252 | 0.0086 | 0.0026 | 0.4187 | 0.1576 | 0.2281 | 0.1561 | 0.8794 |
| **Turicibacter** | 0.0023 | 0.1648 | 0.7956 | 0.0042 | 0.9959 | 0.1140 | 0.0001 | 0.9197 | 0.0065 | 0.0001 | 0.0080 | 0.0053 | 0.8671 | 0.2123 | 0.5056 | 0.0011 | 0.4619 | 0.1097 | 0.0003 | 0.0005 | 0.3704 | 0.1753 | 0.3490 | 0.0386 |
| **Enterococcus** | 0.2356 | 0.3853 | 0.0082 | 0.2283 | 0.0068 | 0.0381 | 0.1179 | 0.9167 | 0.4827 | 0.0071 | 0.3419 | 0.0561 | 0.0043 | 0.0307 | 0.0016 | 0.6217 | 0.0021 | 0.6361 | 0.0082 | 0.3260 | 0.1972 | 0.1599 | 0.0707 | 0.6904 |
| **Lactobacillus** | 0.0225 | 0.0019 | 0.0000 | 0.0108 | 0.0000 | 0.0000 | 0.9384 | 0.0234 | 0.1571 | 0.0573 | 0.0710 | 0.1883 | 0.5610 | 0.0004 | 0.0000 | 0.7859 | 0.0008 | 0.4012 | 0.0633 | 0.8079 | 0.4278 | 0.2186 | 0.1554 | 0.6923 |
| **Staphylococcus** | 0.0515 | 0.0261 | 0.0022 | 0.0119 | 0.0009 | 0.0008 | 0.0795 | 0.0566 | 0.0416 | 0.0004 | 0.0156 | 0.1000 | 0.0693 | 0.0051 | 0.0000 | 0.7228 | 0.0000 | 0.7884 | 0.0005 | 0.0911 | 0.2398 | 0.0373 | 0.0308 | 0.2314 |
| **Clostridia UCG 014 ge** | 0.0000 | 0.0002 | 0.0214 | 0.0000 | 0.0111 | 0.0003 | 0.0003 | 0.3436 | 0.0010 | 0.0000 | 0.0021 | 0.0350 | 0.9120 | 0.9197 | 0.3099 | 0.0120 | 0.8136 | 0.0619 | 0.0001 | 0.0036 | 0.5003 | 0.5779 | 0.5454 | 0.3604 |
| **Clostridium sensu stricto 1** | 0.6880 | 0.1767 | 0.0003 | 0.6760 | 0.0001 | 0.0210 | 0.1778 | 0.3602 | 0.6003 | 0.9257 | 0.8365 | 0.0014 | 0.1749 | 0.0001 | 0.0000 | 0.0366 | 0.0001 | 0.9993 | 0.7526 | 0.5991 | 0.0630 | 0.0286 | 0.0694 | 0.0452 |
| **Blautia** | 0.6528 | 0.1375 | 0.0132 | 0.8984 | 0.0213 | 0.1440 | 0.2107 | 0.7805 | 0.8488 | 0.8669 | 0.7138 | 0.0028 | 0.2026 | 0.0006 | 0.0001 | 0.0709 | 0.0000 | 0.8386 | 0.5815 | 0.0422 | 0.0694 | 0.0941 | 0.2348 | 0.4381 |
| **GCA 900066575** | 0.1952 | 0.0089 | 0.1248 | 0.0286 | 0.2012 | 0.0519 | 0.8825 | 0.0003 | 0.0003 | 0.0277 | 0.0004 | 0.0005 | 0.0004 | 0.1674 | 0.2065 | 0.0006 | 0.0775 | 0.3035 | 0.7269 | 0.0054 | 0.0331 | 0.2145 | 0.0554 | 0.0298 |
| **Lachnoclostridium** | 0.9925 | 0.8490 | 0.4010 | 0.3754 | 0.5397 | 0.7224 | 0.8165 | 0.1638 | 0.3784 | 0.4735 | 0.2093 | 0.0029 | 0.0012 | 0.1717 | 0.0404 | 0.0344 | 0.0257 | 0.7325 | 0.3552 | 0.9493 | 0.1345 | 0.2181 | 0.4057 | 0.8119 |
| **Lachnospiraceae NK4A136 group** | 0.0434 | 0.0669 | 0.5755 | 0.0019 | 0.5289 | 0.0108 | 0.0852 | 0.5310 | 0.1702 | 0.2576 | 0.1425 | 0.0134 | 0.3189 | 0.1498 | 0.2062 | 0.0385 | 0.0519 | 0.2280 | 0.1677 | 0.1517 | 0.0733 | 0.1982 | 0.2624 | 0.7800 |
| **Lachnospiraceae UCG 006** | 0.0001 | 0.0000 | 0.0465 | 0.0000 | 0.1025 | 0.0003 | 0.0160 | 0.0096 | 0.0000 | 0.0000 | 0.0000 | 0.0000 | 0.0301 | 0.0880 | 0.3166 | 0.0000 | 0.0776 | 0.6175 | 0.0020 | 0.0000 | 0.0535 | 0.1094 | 0.0628 | 0.1122 |
| **Lachnospiraceae unclassified** | 0.0050 | 0.0046 | 0.0000 | 0.0008 | 0.0000 | 0.0000 | 0.2696 | 0.0705 | 0.0275 | 0.0207 | 0.0179 | 0.8999 | 0.9249 | 0.0152 | 0.0006 | 0.5297 | 0.0013 | 0.7514 | 0.0012 | 0.4136 | 0.2126 | 0.2863 | 0.4168 | 0.3433 |
| **Marvinbryantia** | 0.1629 | 0.0197 | 0.0012 | 0.1516 | 0.0050 | 0.0014 | 0.5036 | 0.0149 | 0.0942 | 0.2489 | 0.0982 | 0.4790 | 0.4945 | 0.0076 | 0.0009 | 0.6945 | 0.0036 | 0.5720 | 0.5609 | 0.5081 | 0.6925 | 0.5315 | 0.7392 | 0.7304 |
| **Roseburia** | 0.0211 | 0.0164 | 0.0143 | 0.0017 | 0.0041 | 0.0027 | 0.1123 | 0.1177 | 0.0003 | 0.0024 | 0.0007 | 0.0034 | 0.0573 | 0.8292 | 0.8971 | 0.0008 | 0.7338 | 0.8440 | 0.0030 | 0.0078 | 0.9513 | 0.9870 | 0.6987 | 0.9905 |
| **Lachnospiraceae uncultured** | 0.5654 | 0.2323 | 0.0006 | 0.3588 | 0.0024 | 0.0172 | 0.3316 | 0.0118 | 0.2177 | 0.4837 | 0.1034 | 0.6800 | 0.1670 | 0.0002 | 0.0038 | 0.7993 | 0.0056 | 0.8242 | 0.5334 | 0.7316 | 0.8324 | 0.9568 | 0.6150 | 0.5050 |
| **Colidextribacter** | 0.0829 | 0.2124 | 0.6755 | 0.2804 | 0.8089 | 0.6310 | 0.0202 | 0.2277 | 0.5143 | 0.0433 | 0.6269 | 0.9675 | 0.0296 | 0.5000 | 0.7824 | 0.6258 | 0.8858 | 0.2741 | 0.0751 | 0.1361 | 0.7407 | 0.8752 | 0.9074 | 0.1277 |
| **Oscillospiraceae uncultured** | 0.3634 | 0.6198 | 0.6099 | 0.9760 | 0.8263 | 0.7006 | 0.2788 | 0.0502 | 0.8286 | 0.3089 | 0.7193 | 0.4141 | 0.0093 | 0.9249 | 0.5562 | 0.6606 | 0.6428 | 0.8205 | 0.2784 | 0.7609 | 0.1686 | 0.1580 | 0.0985 | 0.7406 |
| **Oscillospirales ge** | 0.0101 | 0.0002 | 0.0000 | 0.0039 | 0.0000 | 0.0000 | 0.7649 | 0.0985 | 0.0282 | 0.0009 | 0.0183 | 0.3995 | 0.3224 | 0.0008 | 0.0000 | 0.6868 | 0.0002 | 0.8212 | 0.0001 | 0.1553 | 0.1277 | 0.0605 | 0.0633 | 0.1617 |
| **Ruminococcus** | 0.0878 | 0.2309 | 0.5881 | 0.0044 | 0.4913 | 0.0319 | 0.0480 | 0.0077 | 0.0003 | 0.0085 | 0.0002 | 0.0031 | 0.0368 | 0.7302 | 0.6196 | 0.0009 | 0.6810 | 0.3528 | 0.4738 | 0.0201 | 0.3219 | 0.6172 | 0.5944 | 0.2613 |
| **Peptococcaceae uncultured** | 0.0772 | 0.0014 | 0.0000 | 0.0468 | 0.0000 | 0.0001 | 0.3690 | 0.0234 | 0.1151 | 0.0478 | 0.0735 | 0.2576 | 0.7046 | 0.0009 | 0.0002 | 0.9581 | 0.0023 | 0.7143 | 0.0678 | 0.4284 | 0.5963 | 0.2696 | 0.6326 | 0.7739 |
| **Romboutsia** | 0.7472 | 0.7090 | 0.1286 | 0.9959 | 0.1466 | 0.5042 | 0.2274 | 0.2611 | 0.9549 | 0.5163 | 0.7844 | 0.4392 | 0.4332 | 0.5080 | 0.3537 | 0.5270 | 0.3727 | 0.3893 | 0.2876 | 0.3869 | 0.7778 | 0.8414 | 0.5547 | 0.6876 |
| **Escherichia Shigella** | 0.0598 | 0.8218 | 0.2461 | 0.0910 | 0.1548 | 0.8473 | 0.0241 | 0.6046 | 0.0561 | 0.0420 | 0.1436 | 0.0015 | 0.1174 | 0.0025 | 0.0305 | 0.0027 | 0.0322 | 0.7267 | 0.4518 | 0.0036 | 0.2223 | 0.1300 | 0.1107 | 0.0326 |
| **Akkermansia** | 0.5201 | 0.0432 | 0.0004 | 0.4697 | 0.0005 | 0.1839 | 0.0003 | 0.3311 | 0.4472 | 0.4843 | 0.5534 | 0.0356 | 0.8629 | 0.0106 | 0.0179 | 0.0910 | 0.0615 | 0.2145 | 0.2938 | 0.1574 | 0.2973 | 0.0736 | 0.0903 | 0.0855 |

**Table S2 adjusted p-values of correlations with phenotypic outcomes**
