# Supplementary material for: A novel ketogenic diet that reduces seizures and prevents liver steatosis leads to related gut microbiome changes and restores cecal short-chain fatty acid levels in the rapid kindling rat model of epileptogenesis
Source: Gut Microbes Rep. 2025 Oct 9;2(1):2567677. doi: 10.1080/29933935.2025.2567677 (PMC12899332; doi:10.1080/29933935.2025.2567677)
Supplement: Supplementary material [file KGMR_A_2567677_SM7717.docx]

# Supplemental material

**Figure S1 hierarchical clustering including the non-kindled groups**

**Figure S2** **Microbiota metabolites and cecum pH among all groups**

Box and whisker plots of the cecum median total SCFA (A), acetate (B), propionate (C), butyrate (D), iso butyrate (E), valerate (F) and iso valerate levels (G), the resulting butyrate to propionate molar ratio (H), Box and whisker plots of ammonia levels (I) and pH values (J). Metabolite values are expressed in mmol/kg and values below the detection range are set to the lowest limit of detection and were excluded from the butyrate to propionate ratio analysis. Dots represent outliers. Significance was assessed by pairwise Wilcoxon test, * *P* < 0.05, ** *P* < 0.01, *** *P* < 0.001, **** *P* < 0.0001.

**Table S1 correlation coefficients of correlations with phenotypic outcomes**

**Table S2 adjusted p-values of correlations with phenotypic outcomes**
